# Biological aging and generational shifts in early-onset cancer risk

**DOI:** 10.1038/s41591-026-04448-w

**Published:** 2026-06-22

**Authors:** Ruiyi Tian, Xiaoyu Zong, Duo Ren, Stefani Tica, Daniel Hong, Oluseye Oduyale, Jason D. Buenrostro, Ramaswamy Govindan, Yin Cao

**Affiliations:** 1 Department of Surgery, Division of Public Health Sciences, Washington University School of Medicine in St. Louis, St. Louis, MO USA; 2 Alvin J. Siteman Cancer Center, Washington University School of Medicine in St. Louis, St. Louis, MO USA; 3 Department of Pediatrics, Division of Gastroenterology, Hepatology & Nutrition, Washington University School of Medicine in St. Louis, St. Louis, MO USA; 4 Department of Radiation Oncology, Washington University School of Medicine in St. Louis, St. Louis, MO USA; 5 Department of Surgery, Washington University School of Medicine in St. Louis, St. Louis, MO USA; 6 Department of Stem Cell and Regenerative Biology, Harvard University, Cambridge, MA USA; 7 Biology of Adversity Project, Broad Institute of MIT and Harvard, Cambridge, MA USA; 8 Department of Medicine, Division of Oncology, Washington University School of Medicine in St. Louis, St. Louis, MO USA; 9 Department of Medicine, Division of Gastroenterology, Washington University School of Medicine in St. Louis, St. Louis, MO USA; 10 Institute for Informatics, Data Science and Biostatistics, Washington University School of Medicine in St. Louis, St. Louis, MO USA

**Keywords:** Cancer epidemiology, Risk factors

## Abstract

Incidence of early-onset cancer is rising globally in recent generations, which underscores the need to elucidate the influence of emerging generational risk factors. Systemic and organ-specific aging reflects the cumulative impact of exposures and may provide an integrative and complementary approach to understand early-onset cancer risk. Here among 154,169 young adults from the United Kingdom Biobank, systemic aging measured by PhenoAge increased across birth cohorts, with 23% s.d. increase for those born 1965–1974 versus 1950–1954, and was associated with early-onset solid cancer risk (hazard ratio (HR)_per s.d._ 1.08; 95% confidence interval (CI), 1.03–1.13), driven by lung, gastrointestinal and uterine cancers, independent of genetic risks of aging and cancer. Patterns were consistent using alternative systemic aging measures, including the Klemera–Doubal method-defined age gap and metabolomic-based age gap. These findings were validated partially among 10,262 participants in the United States All of Us Research Program. Proteomics-based organ-specific aging analyses linked immune aging with early-onset lung cancer (HR_per s.d._ 1.89; CI, 1.20–2.97) and adipose tissue aging to early-onset colorectal cancer (HR 1.60; CI, 1.11–2.32). Greater age gap, reflecting more advanced biological aging relative to chronological age, may serve as a driver associated with risk of early-onset solid cancers, highlighting the importance of uncovering underlying mechanisms to guide effective prevention strategies.

## Abstract (web summary)

Analyses of population cohorts found that young adults exhibited earlier systemic and organ-specific aging, which was associated with increased risk of early-onset cancer compared with older adults born decades earlier.

## Main

Over the past three decades, early-onset cancers, diagnosed in adults often under age 50 or 55 years, have become a global cancer prevention and public health challenge^1–9^. Between 1990 and 2019, cancers diagnosed under the age of 50 years increased by 24% globally and continue to rise^4^. In the United States (US), this increasing trend is led by several cancers, including multiple myeloma, colorectal cancer and uterine cancer^1,5^. Notably, this upward trend is more pronounced in recent generations compared to earlier ones in many countries^3,5,6,8^. In Australia, Canada, the United Kingdom (UK) and the US, people born in the 1990s face at least a fourfold higher risk of early-onset colorectal cancer compared with those born in the 1960s^6^. In the US, compared with people born before 1950, those born circa 1985 have approximately twice the risk of uterine cancer^1^. Moreover, cohorts with elevated incidence before age 50 years seem to carry this excess risk into ages 50–54 years. In the US, the proportion of colorectal cancers diagnosed before age 55 increased from 11% in 1995 to 20% in 2019^3^, and in the UK, incidence among adults aged 50–54 years rose by 0.58% annually between 2008 and 2017 ^6^. Together, these patterns suggest the influence of emerging generational risk factors.

Identifying the full set of risk factors for early-onset cancers remains a research priority, yet is challenging. Many relevant exposures are measured incompletely across the life-course, individual effects may be modest and etiologic drivers probably occur as mixtures that co-occur and interact. In this context, measurements that integrate cumulative exposure and capture shared biology across several cancers may provide a complementary strategy^10,11^. Aging, which reflects the cumulative impact of exposures and intersects with several cancer hallmarks^12^, may represent a candidate measure^13^. A wide array of physiological and environmental factors can converge on aging-related processes, including chronic inflammation, cumulative genetic damage, epigenetic changes, alterations in the tissue microenvironment, and dysregulation of adaptive and innate immunity, all of which have been linked to processes involved in tumor initiation and progression^10^. These processes can reinforce one another, reshaping both systemic and organ-specific tissue contexts^14^ and increasing susceptibility to malignant transformation, ultimately accelerating cancer onset at younger ages. Furthermore, given that people’s aging trajectories may vary across organ systems and diverge from systemic aging of the whole body^14^, organ-specific aging may influence early-onset cancer risk either independently or in concert with systemic aging.

However, prospective epidemiological evaluation of both systemic and organ-specific aging in early-onset cancers remains limited. In addition, whether newer generations experience a greater age gap, defined here as people exhibiting biological profiles that seem older than expected for their chronological age^14,15^ relative to previous birth cohorts remains uncharacterized. Secular shifts such as earlier puberty^16^ and earlier onset of obesity, metabolic syndrome, diabetes and stroke^17^, all point toward greater accelerated aging in recent birth cohorts. Given that many of these conditions either contribute to or act as risk factors for cancer, a better understanding of how generational differences in accelerated aging relate to early-onset cancers is needed urgently.

**Fig. 1:**
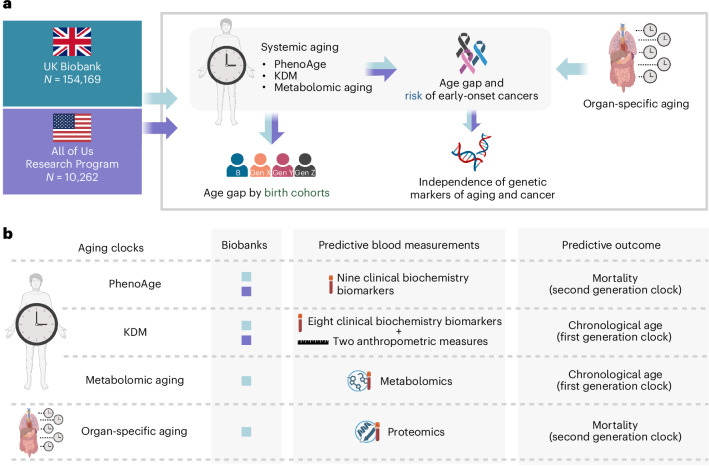
Study design and overview of aging clocks. **a**, Study diagram: systemic aging in UK Biobank and All of Us Research Program, was compared across birth cohorts and linked prospectively with risk of early-onset solid cancers. Furthermore, organ-specific aging and its associations with risk of early-onset solid cancers were assessed in the UK Biobank. **b**, Summary of biological aging clocks used in this study, including predictive measurements and predictive outcomes used for each clock. Figure created in BioRender; Cao, Y. https://biorender.com/lbrmz17 (2026). KDM, Klemera-Doubal method.

As early-onset cancers are relatively uncommon, these questions require large, well-phenotyped cohorts with long follow-up. Such resources are only now becoming available, with harmonized clinical biochemistry and multi-omics profiles, particularly metabolomics and proteomics. As biological aging can be quantified using complementary clocks that each capture partially distinct dimensions of aging biology^18^, and cancer has emerged as a systemic disease^10^, we conducted an observational cohort study leveraging data available in a large UK cohort (UK Biobank) and an electronic health record (EHR)-linked biobank in the US (All of Us Research Program), first prioritizing integrative measures of systemic aging and then evaluating organ-specific aging (Fig. 1). Specifically, we applied the clinical measurement-based (PhenoAge^15^ and Klemera–Doubal method (KDM)^19^) and metabolomic-based^20^ systemic biological aging algorithms to characterize birth-cohort patterns of accelerated aging and their prospective associations with early-onset cancers independently of genetic markers of aging and cancer susceptibility. We then complemented these analyses with proteomics-based organ-specific aging clocks^14^ to delineate the independent contributions of systemic and organ-specific aging to early-onset cancer risk.

## Results

### UK Biobank

#### Systemic aging across birth cohorts

**Fig. 2:**
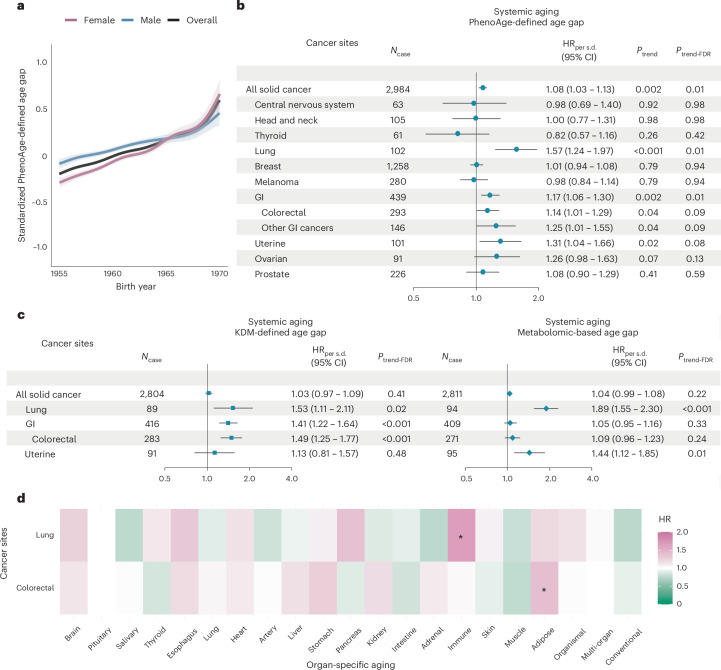
Systemic and organ-specific aging across birth cohorts and associations with early-onset solid cancer risk, UK Biobank (2006–2021). **a**, In the UK Biobank, GAMs were used to depict the relationship between systemic aging (standardized PhenoAge-defined age gap) and birth year, adjusted for age and age^2^ (details in Supplementary Table 1). Lines: model-estimated means; shaded bands: 95% CIs. Higher values indicate greater mortality and morbidity risk. **b**, Associations between systemic aging (PhenoAge-defined age gap) and risk of early-onset solid cancers (<55 years) estimated using Cox proportional hazards models. Points represent HR_per s.d._ increase in PhenoAge-defined age gap, adjusted for sex, race, Townsend Deprivation Index, education, BMI, smoking status and intensity, alcohol intake per day, diet, physical activity, personal history of chronic obstructive pulmonary disease, cardiovascular disease and diabetes, and the first ten PCs of genetic ancestry. Horizontal lines: 95% CIs; vertical line: HR = 1. Two-sided Wald tests were used to derive *P* values. *P* values for trend (*P*_trend_) are reported in Extended Data Table 2, and FDR-adjusted *P* values (Benjamini–Hochberg; *P*_trend-FDR_) are shown in the figure. *P*_trend_ for early-onset lung cancer is 1.3 × 10^−4^. **c**, Validation analyses using alternative systemic aging measures, including KDM-defined and metabolomic-based, demonstrating directionally consistent associations for early-onset lung and GI cancers. Squares and diamonds: HR_per s.d._ increase in age gap; horizontal lines: 95% CIs; vertical line: HR = 1. Two-sided Wald tests were used to derive *P* values. *P* values for trend (*P*_trend_) were reported in Supplementary Tables 5 and 7. Multiple comparisons were controlled using the Benjamini–Hochberg procedure, with adjusted *P* values shown (*P*_trend-FDR_). *P*_trend-FDR_ for KDM-defined age gap and early-onset GI cancers is 1.7 × 10^−5^; *P*_trend-FDR_ for KDM-defined age gap and early-onset colorectal cancer is 1.7 × 10^−5^; *P*_trend-FDR_ for metabolomic-based age gap and early-onset lung cancer is 1.25 × 10^−9^. **d**, Heatmap of HRs evaluating associations between proteomic-based organ-specific aging measures and early-onset lung and colorectal cancer. Two-sided Wald tests were used to derive *P* values, and multiple comparisons were controlled using the Benjamini–Hochberg procedure. Black stars: statistically significant associations (*P*_FDR_ < 0.05). Organismal aging includes proteins broadly expressed across organs; multi-organ aging includes all organ-specific markers; conventional aging includes all plasma proteins. BMI, body mass index; CI, confidence interval; FDR, false discovery rate; GAM, generalized additive model; GI, gastrointestinal; HR, hazard ratio; KDM, Klemera-Doubal method; PC, principal component; s.d., standard deviation.

**Extended Data Fig. 1:**
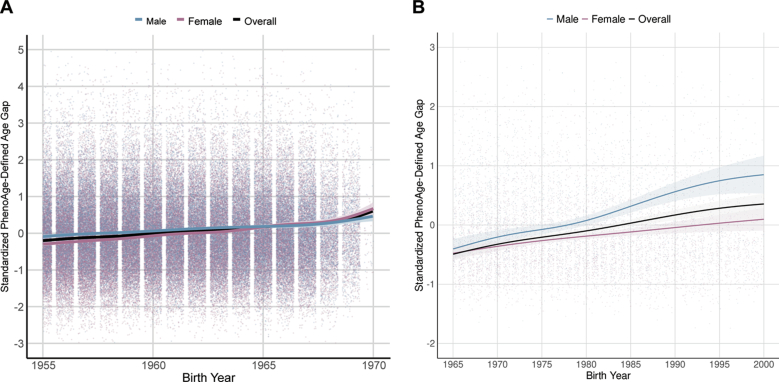
Birth cohort trends in systemic age gap in UK Biobank (2006-2021) and All of Us Research Program (2010-2022). Generalized additive models depicting the relationship between PhenoAge-defined age gap and birth year, adjusted for age and age^2^ in **a**, UK Biobank (n = 154,169) **b**, All of Us Research Program (n = 10,262). Each point represents one individual. Blue and red points/lines denote males and females, respectively; black lines indicate the overall population. Each data point represents one individual (biological replicate). Solid lines represent fitted values from generalized additive models (GAMs) with smoothing splines, and shaded areas indicate 95% confidence intervals.

**Extended Data Fig. 2:**
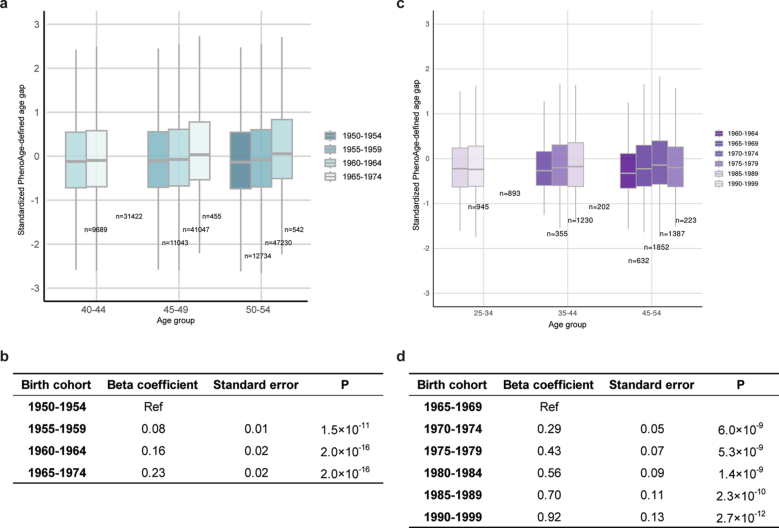
Birth-cohort differences in PhenoAge-defined age gap in UK Biobank (2006-2021) and All of Us Research Program (2010-2022). **a**, Distribution of standardized PhenoAge-defined age gap across birth cohorts within age groups (40–44, 45–49, and 50–54 years) in the UK Biobank. Box plots show the median (center line), IQR (box), and whiskers extending to 1.5×IQR. Sample sizes (n) for each group are indicated below the x axis. Statistical comparisons between adjacent birth cohorts were performed using pairwise two-sided t-tests with Bonferroni correction for multiple comparisons. Exact adjusted P values are as follows: age group 45–49 years, P_1955-1959/1960-1964_ = 2.2×10^−4^ and P_1960-1964/1965-1974_ = 0.00739; age group 45–49 years, P_1950-1954/1955-1959_ = 7.9×10^−8^ and P_1955-1959/1960-1964_ = 0.00133. **b**, Linear regression estimates of the association between birth cohort and PhenoAge-defined age gap in the UK Biobank, with the earliest birth cohort as the reference group and adjustment for age and age^2^. Beta coefficients represent differences relative to the reference cohort (1950–1954). **c**, Distribution of standardized PhenoAge-defined age gap across birth cohorts within age groups (25–34, 35–44, 45–54 years) in the All of Us Research Program. Box plots show the median (center line), IQR (box), and whiskers extending to 1.5×IQR. Sample sizes (n) for each group are indicated below the x axis. Statistical comparisons between adjacent birth cohorts were performed using pairwise two-sided t-tests with Bonferroni correction for multiple comparisons. Exact adjusted P values are as follows: age group 35–44 years, P_1970-1974/1985-1989_ = 0.04; age group 45–54 years, P_1960-1964/1965-1969_ = 0.0022, P_1960-1964/1970-1974_ = 1.0×10^−6^. **d**, Linear regression estimates of the association between birth cohort and PhenoAge-defined age gap in the All of Us Research Program, with the earliest birth cohort as the reference group and adjustment for age and age^2^. Beta coefficients are presented relative to the reference cohort (1965–1969) from multivariable linear regression models. Abbreviation: IQR, interquartile range.

Among 154,169 UK Biobank participants (55% female, 92% White) analyzed for birth-cohort-specific trends (Table 1), later cohorts had progressively higher levels of PhenoAge-defined age gap (Figs. 1 and 2a, Extended Data Figs. 1a and 2a and Supplementary Table 1). Male participants born before 1965 showed higher baseline levels, but female participants experienced a steeper increase over time (Fig. 2a; *P*_interaction_ < 0.001; *P*_likelihood ratio test_ < 0.001). Compared to those born in 1950–1954, people born in 1965–1974 had a 23% higher (s.e. 0.02) standardized PhenoAge-defined age gap (Extended Data Fig. 2b).

**Table 1:** Baseline characteristics of 154,169 participants from UK Biobank (2006–2021) and 10,262 participants from All of Us Research Program (2010–2022)

*N*	UK Biobank	All of Us Research Program
	154,169	10,262
Age at baseline/enrollment, year, mean (s.d.)	48.3 (4.2)	41.6 (10.3)
Mean age between first and last measurements, year, mean (s.d.)		40.3 (10.3)
Years between first and last measurements, mean (s.d.)		1.3 (1.5)
Years between first measurement and enrollment, mean (s.d.)		1.9 (2.0)
Female, *n* (%)	84,466 (55%)	7,034 (69%)
White, *n* (%)	141,330 (92%)	
NHW, *n* (%)		5,488 (54%)
NHB, *n* (%)		1,688 (16%)
Hispanic, *n* (%)		2,485 (24%)
Townsend Deprivation Index, mean (s.d.)	−1.0 (3.2)	
Social Deprivation Index, mean (s.d.)		0.3 (0.1)
Postcollege, *n* (%)	67,861 (44%)	6,871 (67%)
BMI, kg m^−2^, mean (s.d.)	27.2 (4.9)	31.7 (9.3)
Smoking status, *n* (%)		
Never	93,169 (60%)	6,894 (67%)
Past	40,889 (26%)	1,957 (19%)
Current	20,364 (13%)	1,411 (14%)
Alcohol consumption, *n* (%)		
Never	6,463 (4.2%)	1,216 (12%)
Past	4,962 (3.2%)	1,802 (18%)
Current	142,997 (93%)	7,244 (71%)
Healthy diet, *n* (%)	67,950 (44%)	
Family history of cancer, *n* (%)	36,900 (24%)	1,633 (16%)

Continuous variables are presented as mean (s.d.) and categorical variables are presented as number (percentage). Healthy diet was defined as meeting at least four of seven recommended dietary components for cardiometabolic health, based on available 24-h recall and FFQ data (fruit and vegetable intake, whole grains, red and processed meat, fish and moderate alcohol intake). BMI, body mass index; FFQ, Food Frequency Questionnaire; NHB, non-Hispanic Black; NHW, non-Hispanic White; s.d., standard deviation.

#### Systemic aging with risk of early-onset cancers

**Extended Data Fig. 3:**
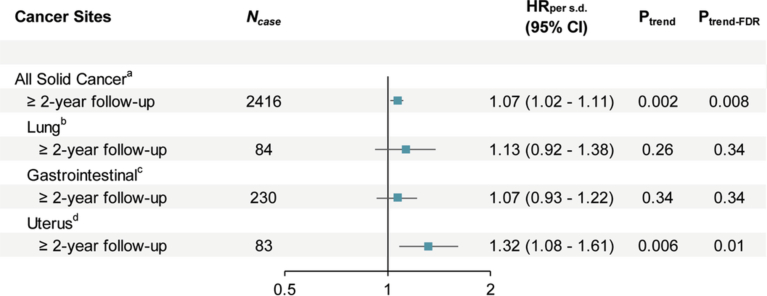
Associations between PhenoAge-defined age gap and risk of all solid cancer, lung, gastrointestinal, and uterine cancer, according to follow-up length, UK Biobank 2006-2021. Cox proportional hazards regression model outputs for the association between PhenoAge-defined age gap and early-onset solid cancers by follow-up length. Squares represent: HR_per s.d._ increase in age gap; horizontal lines: 95% CIs; vertical line: HR = 1. Two-sided Wald tests were used to derive P values, and multiple comparisons were controlled using the Benjamini-Hochberg procedure. All models adjusted for age at baseline, sex, race, the Townsend Deprivation Index, education, BMI, smoking status and intensity, alcohol intake per day, diet, personal history of chronic obstructive pulmonary disease, cardiovascular disease, and diabetes, and the first 10 PCs of genetic ancestry. **a**. Additionally adjusted for family history of lung, breast, colorectal, and prostate cancers. **b**. Additionally adjusted for family history of lung cancers. **c**. Additionally adjusted for family history of colorectal cancers. **d**. Calculated among female participants only and additionally adjusted for age at menarche, oral contraceptive use, menopause, and parity. P_trend-FDR_ was calculated across the five cancer site-specific tests presented in this figure. Abbreviations: BMI, body mass index; CI, confidence interval; FDR, false discovery rate; HR, hazard ratio; PC, principal component; s.d., standard deviation.

**Extended Data Fig. 4:**
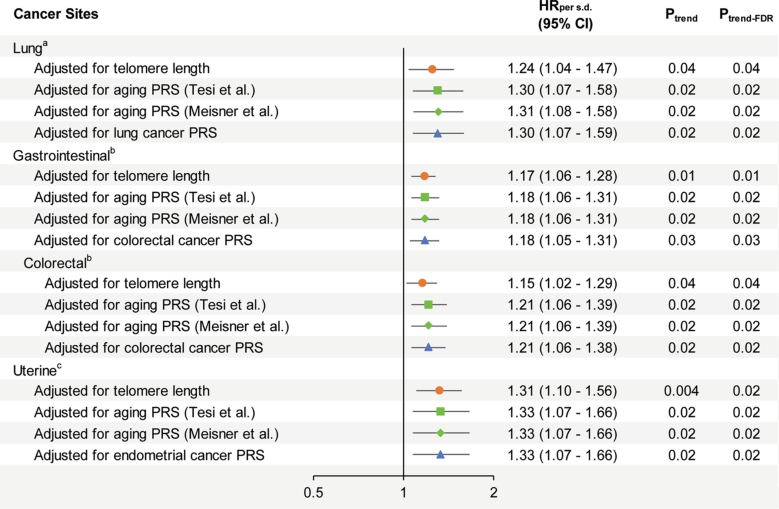
Associations between PhenoAge-defined age gap and early-onset lung, gastrointestinal and uterine cancers, additionally adjusted for genetic markers of aging and cancer, UK Biobank (2006-2021). Cox proportional hazards regression model outputs for the association between PhenoAge-defined age gap and early-onset solid cancers after additionally adjusting for leukocyte telomere length, aging, and cancer-specific PRSs. PRS related analysis were performed in 105,628 participants after quality control. Two aging PRS were used, including one focusing on longevity (Tesi et al.) and one focusing on lifespan modified by non-genetic risk factors (Meisner et al.). Circles, squares, diamonds and triangles denote models adjusted for telomere length, aging PRS (Tesi et al.), aging PRS (Meisner et al.), and site-specific cancer PRS, respectively. Horizontal lines: 95% CIs; vertical line: HR = 1. Two-sided Wald tests were used to derive P values, and multiple comparisons were controlled using the Benjamini-Hochberg procedure. All models adjusted for age at baseline, sex, race, the Townsend Deprivation Index, education, BMI, smoking status and intensity, alcohol intake per day, diet, personal history of chronic obstructive pulmonary disease, cardiovascular disease, and diabetes, and the first 10 principal components of genetic ancestry. **a**. Additionally adjusted for family history of lung cancer. **b**. Additionally adjusted for family history of colorectal cancer. **c**. Calculated among female participants only and additionally adjusted for age at menarche, oral contraceptive use, menopause, and parity. Abbreviation: BMI, body mass index; CI, confidence interval; FDR, false discovery rate; HR, hazard ratio; PRS, polygenic risk score; s.d., standard deviation.

Over 953,582 person-years of follow-up of 148,317 participants (Extended Data Table 1), PhenoAge-defined age gap was associated with an increased risk of early-onset solid cancers (Fig. 2b and Extended Data Table 2). Compared with those in the lowest tertile, people in the highest tertile had a 1.15-fold higher risk for early-onset solid cancers (HR 1.15; 95% CI, 1.03–1.28) (Extended Data Table 2). Each s.d. increment in PhenoAge-defined age gap was associated with an 8% increased risk of early-onset solid cancers (HR_per s.d._ 1.08; 95% CI, 1.03–1.13), driven mainly by lung (HR_per s.d._ 1.57; 95% CI, 1.24–1.97), gastrointestinal (GI) (HR_per s.d._ 1.17; CI, 1.06–1.30 for overall; HR 1.14; CI, 1.01–1.29 for colorectal; HR 1.25; CI, 1.01–1.55 for other GI cancers) and uterine (HR_per s.d._ 1.31; CI, 1.04–1.66) cancers (Fig. 2b; all *P*_trend_ < 0.05). In contrast, the associations were weaker for cancers diagnosed after age 55 years (Extended Data Table 3). These findings remain similar after excluding participants with less than 2 years of follow-up (Extended Data Fig. 3), after additional adjustment for leukocyte telomere length and genetic predisposition to aging or cancer (Extended Data Fig. 4), and when redefining early-onset cancers using <50 years as cutoff (Supplementary Table 2). Mediation analyses show that PhenoAge-defined age gap mediates only modestly the association between lifestyle risk factors and early-onset solid cancers (Supplementary Table 3).

To complement mortality- and morbidity-based systemic aging measure (PhenoAge) with chronological-age-trained clocks, we also examined systemic aging using the KDM among 137,553 participants in the UK Biobank (Supplementary Table 4), KDM-defined age gap showed directionally similar but weaker associations with early-onset solid cancers overall (HR_per s.d._ 1.03; 95% CI, 0.97–1.09; *P*_trend_ = 0.33) (Fig. 2c and Supplementary Table 5). However, KDM-defined age gap was associated with increased risks of early-onset lung (HR_per s.d._ 1.53; CI, 1.11–2.11) and GI cancers (HR_per s.d._ 1.41; CI, 1.22–1.64).

To fully leverage available multi-omics data to assess systemic aging, we leveraged metabolomic aging clocks and evaluated their association with early-onset solid cancers among 140,373 participants (Supplementary Table 6). Increased metabolomic-based age gap was associated modestly with early-onset solid cancers overall (HR_per s.d._ 1.04; 95% CI, 0.99–1.08; *P*_trend_ = 0.13), driven by lung cancer (HR_per s.d._ 1.89; 95% CI, 1.55–2.30) and uterine cancer (HR_per s.d._ 1.44, CI, 1.12–1.85), whereas associations with GI cancer and colorectal cancer were not statistically significant (Fig. 2c and Supplementary Table 7).

#### Organ-specific aging with risk of early-onset cancers

To identify the key organ sites contributing to early-onset solid cancer risk, we performed plasma proteomics-based organ-specific aging analyses^14^ among 19,874 participants in the UK Biobank. Immune tissue aging was associated with early-onset lung cancer (HR_per s.d._ 1.89; 95% CI, 1.20–2.97), and adipose tissue aging was associated with increased risk of early-onset colorectal cancer (HR 1.60; CI, 1.11–2.32) (Fig. 2d and Supplementary Table 8). These associations remained robust after additional adjustment for systemic aging (immune-lung cancer: HR 1.85; CI, 1.17–2.93; adipose-colorectal cancer: HR 1.60; CI, 1.11–2.32) (Supplementary Table 8), supporting the probably independent contribution of organ-specific aging to early-onset cancer development.

### All of Us Research Program

#### Systemic aging across birth cohorts

**Fig. 3:**
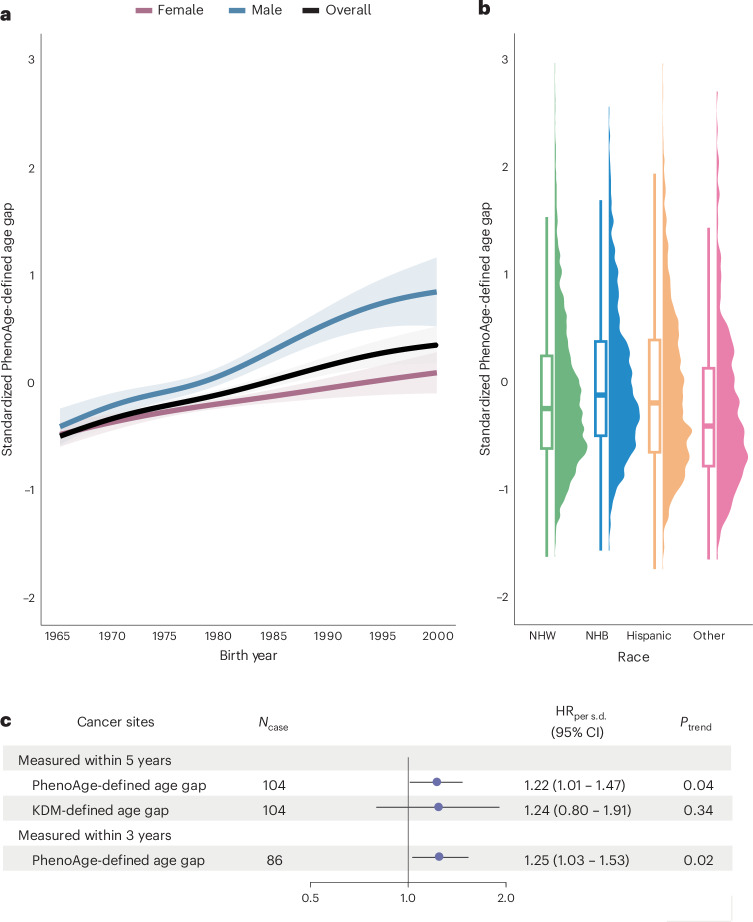
Systemic aging across birth cohorts and associations with early-onset solid cancer risk, All of Us Research Program (2010–2022). **a**, GAM depicting the relationship between systemic aging (PhenoAge-defined age gap) and birth year, adjusted for age and age^2^ (details in Supplementary Table 1). Lines: model-estimated means; shaded bands: 95% CIs. Higher values indicate greater mortality and morbidity risk. **b**, Systemic aging (PhenoAge-defined age gap) by race/ethnicity: NHW (*n* = 5,488), NHB (*n* = 1,688), Hispanic (*n* = 2,485) and Other (*n* = 601). Violin plots: distribution density; embedded box plots: median (center line) and IQR (box); whiskers extend to 1.5× IQR. Pairwise comparisons were performed using two-sided *t*-tests. Exact *P* values are as follows: NHB versus NHW, P = 1.7 × 10^−7^; Hispanic versus NHW, P = 5.4 × 10^−9^; NHB versus Other, P = 1.3 × 10^−6^; Hispanic versus Other, P = 8.2 × 10^−7^; NHW versus Other, P = 0.20. **c**, Associations between systemic aging (PhenoAge and KDM-defined age gap) and risk of early-onset solid cancers (<55 years) estimated using Cox proportional hazards models. Points: HR_per s.d._ increase in PhenoAge and KDM-defined age gap, adjusted for sex, race, the Social Deprivation Index, education, BMI, smoking status, alcohol intake, family history of cancer, personal history of chronic obstructive pulmonary disease, cardiovascular disease and diabetes. Horizontal lines: 95% CIs; vertical line: HR = 1. BMI, body mass index; CI, confidence interval; FDR, false discovery rate; GAM, generalized additive model; HR, hazard ratio; IQR, interquartile range; KDM, Klemera-Doubal method; NHB, non-Hispanic Black; NHW, non-Hispanic White; PC, principal component; s.d., standard deviation.

Among 10,262 participants in the All of Us Research Program (69% female; 54% non-Hispanic White (NHW), 24% Hispanic, 16% non-Hispanic Black (NHB)) (Table 1), PhenoAge-defined age gap similarly increased across birth cohorts (Fig. 3a and Extended Data Fig. 2c). Male participants consistently showed higher levels of PhenoAge-defined age gap than female participants and, compared to those born in 1965–1969 (Fig. 3a), people born in 1990–1999 had a 92% higher standardized PhenoAge-defined age gap (s.e. 0.13) (Extended Data Fig. 2d). NHB and Hispanic participants had higher average levels than NHW participants (*P* < 0.001; Fig. 3b).

#### Systemic aging with risk of early-onset solid cancers

The participants included in the prospective analysis on risk of early-onset solid cancers had similar characteristics to the overall cohort (Extended Data Table 4). Over 14,791 person-years, 104 cases of early-onset solid cancer were identified. Increased PhenoAge-defined age gap was associated prospectively with the risk of early-onset solid cancers, with each s.d. increase in PhenoAge-defined age gap associated with a 22% higher risk (HR 1.22; 95% CI, 1.01–1.47; *P*_trend_ = 0.04; Fig. 3c). Results remained robust when limited to measurements measured within 3 years. KDM-defined age gap showed a directionally consistent but weaker association with early-onset solid cancer risk (Fig. 3c).

## Discussion

In this integrated analysis leveraging UK and US cohort and EHR-linked biobank data, we observed that systemic aging increased across birth cohorts from the 1950s to the 1990s. Notably, systemic aging, measured by age gap using both clinical biochemistry and metabolomics, and defined using mortality- and morbidity-based or chronological-age-based metrics, was associated with a higher risk of early-onset solid cancers, particularly lung, GI and uterine cancers, independent of genetic predisposition to aging and cancer. Exploratory analyses of organ-specific aging, measured by proteomics, revealed selective associations: immune aging linked to early-onset lung cancer, and adipose tissue aging linked to early-onset colorectal cancer, providing site-specific biological validation of the systemic findings. Together, these findings provide population-based evidence that a greater age gap (systemic or organ-specific) may serve as an integrative measurement of physiological dysregulation arising from the cumulative burden of established and emerging risk factors for early-onset cancers in recent generations. If validated, these findings may inform future directions in etiological and preventive research.

To date, few studies have assessed whether systemic aging varies by birth cohort. This question is central because early-onset cancers show strong birth-cohort effects, with more recent generations experiencing higher risk as they age. If systemic aging also shifts across cohorts, it could represent a measurable marker reflecting shared patterns with birth-cohort-specific cumulative exposures associated with rising early-onset cancer risk. Indeed, we observed that more recent birth cohorts exhibit a greater age gap, with modest nonlinear patterns that may reflect the accumulation and interaction of exposures across generations. Emerging evidence suggests that accelerated aging is linked to a broad range of exposomic factors, including physical (air quality), social (socioeconomic and gender inequality, migration) and sociopolitical (representation, party freedom, suffrage, elections and democracy) determinants^21^. In this context, the higher burden in recent generations may be consistent with earlier and more sustained exposures to obesity and metabolic dysfunction, poorer diet quality, prolonged sedentary time and increasingly common exposures such as circadian disruption and pervasive environmental chemicals, along with other uncharacterized factors that collectively alter the systemic biological environment^22^. Clarifying the contributors to these nonlinear patterns will require studies with repeated, longitudinal assessments of aging markers to quantify within-person aging trajectories. Recent longitudinal investigations suggest that within-person change over time captures aging dynamics more accurately than single timepoint cross-sectional estimates^23,24^.

Although our overall findings are in line with previous work linking a greater age gap to cancer risk in older adults^24–26^, evaluating this association earlier in the life-course is both biologically grounded and, if validated, provides a complementary approach in addressing the principal challenges in early-onset cancer etiology: fully enumerating all contributing exposures is difficult. In particular, the association between a greater age gap by mid-adulthood and risk of early-onset cancers is biologically plausible for several reasons. First, it aligns with evolutionary models of carcinogenesis, that cancer risk is driven not solely by the accumulation of mutations but also by age-related physiological decline that alters tissue microenvironments and shifts somatic selection pressures, enabling previously neutral mutations to acquire fitness advantages and expand clonally^27^. Second, a life-course perspective suggests that the aging-related biological consequences are nonlinear. In later life, more advanced biological aging may exert tumor-suppressive effects through loss of stemness, diminished regenerative capacity, and increased cellular senescence^28^. In contrast, earlier in adulthood, before these constraints are fully established, a greater age gap may be associated with biological contexts that are more permissive for tumor initiation and progression at younger ages. Our findings suggested that age gap may be particularly relevant for understanding early-onset cancers with rising incidence, such as colorectal and uterine cancers^1^, as well as for cancers with substantial unexplained risk, such as lung cancer. In the UK, 67% of young lung cancer patients are diagnosed at stage IV^29^, and in the US, the incidence of early-onset lung cancer is now higher among women, who are mostly never-smokers^30,31^. Finally, our analyses showed the associations between age gap and early-onset lung, GI and uterine cancers are independent of telomere length, a known marker of biological age^32^, genetic predisposition of aging^33,34^ as well as cancer^35–37^, suggesting age gap captures risk beyond canonical and inherited aging pathways and motivating additional mechanistic studies.

Cancer sites also differ in their vulnerability to aging-related biological changes. In our analyses, lung cancer emerged as a predominant contributor to the overall association between systemic aging and early-onset solid cancer risk, with associations remaining statistically significant after adjustment for smoking status and cumulative pack-years. This pattern suggests that age gap may capture additional smoking-independent or smoking-residual processes, such as the accumulation of somatic mutations and clonal hematopoiesis^38^, particularly in tissues with limited regenerative capacity, such as the lung^39,40^. GI cancers, including colorectal cancer, may be particularly influenced by aging-related metabolic^41^ and inflammatory changes^42^ that interact with adiposity and the gut microbiome. Uterine cancer susceptibility to aging may be amplified by the endometrium’s pronounced hormonal and metabolic responsiveness^43^. Our organ-specific aging analyses further support these patterns, showing that organ-specific aging metrics capture exposure-linked biological processes aligned with dominant etiologic pathways at specific sites^14^. Specifically, immune aging was associated with early-onset lung cancer, probably reflecting chronic airway inflammation and immune remodeling driven by inhaled exposures (for example, smoking, air pollution) in the lung as a primary target tissue^22^, whereas adipose tissue aging was associated with early-onset colorectal cancer through visceral adipose-gut crosstalk affecting inflammatory, metabolic, and insulin–insulin-like growth factor 1 signaling^41^. Although these site-specific associations require both epidemiologic and mechanistic validations, we cannot exclude modest associations at other sites. In animal models, age-related biological processes exert both tumor-promoting and tumor-suppressive effects, depending on the tissue context and disease stage^44^. Accordingly, analyses of age gap at the population level may average over these opposing influences, which could contribute to weaker or null associations observed for certain cancer sites. Future studies with larger sample sizes for each cancer site, tissue-relevant aging measurements and mechanistic interrogation are warranted.

Our study has several strengths. First, we leveraged two large population- and EHR-linked biobanks from UK and US, which allowed for an integrated prospective evaluation of age gap in relation to early-onset solid cancers. Early-onset cancer started to increase in the 1990s in successive birth cohorts born after 1955^3,4,6,45^. The birth cohorts represented in our study (UK Biobank: born from 1955 onwards; All of Us: born from 1965 onwards) align with the relevant generations, supporting the potential generalizability of our findings to more recent cohorts if validated. Second, we leveraged rich clinical measurement and multi-omics data maximally to characterize both systemic and organ-specific aging, strengthening the robustness and translational relevance of the results. Third, we analyzed aging markers by birth cohort, providing evidence that age gap patterns track with population-level patterns in early-onset cancer incidence.

Several limitations should be noted. Sample size constraints limited cancer site-specific analyses in the All of Us Research Program and organ-specific aging analyses in the UK Biobank. Validations in studies with larger sample sizes and ideally with longitudinal aging measurements of both using systemic- and organ-specific approaches, are warranted. As an observational study, residual confounding cannot be fully ruled out despite extensive adjustment for established risk factors. In addition, although we leveraged two large populations in the UK Biobank and the All of Us Research Program, our findings reflect primarily populations from the UK and the US. As such, generalizability to populations in other countries or with different demographic, socioeconomic, environmental and healthcare contexts may be limited. Finally, although we leveraged widely applied and validated aging clocks, some have limited validation in various populations and in younger adults, which may affect generalizability across settings. As proteomic-based aging models developed by Goeminne and colleagues^14^ were derived from 44,952 participants in the UK Biobank, partial population overlap with our analytic samples on organ-specific aging and early-onset cancers is unavoidable, raising the concern of circularity and information leakage. We applied the published coefficients without retraining the model, which reduces but does not eliminate this concern. Although the original models were trained to predict overall mortality rather than incident early-onset cancer, and we also prioritized early-onset cancers associated with systemic aging, independent validation in external cohorts is warranted to confirm our exploratory findings on organ-specific aging.

In conclusion, prospective UK and US data show rising age gap across birth cohorts, potentially associated with increased risk of several early-onset solid cancers. Uncovering underlying mechanisms and life-course drivers will be critical to inform prevention and early intervention strategies.

## Methods

### UK Biobank

#### Study design and population

For primary discovery, we conducted an observational cohort study leveraging the UK Biobank (Fig. 1a)—a population-based cohort of over 500,000 participants aged 37–73 years between 2006 and 2010, with ongoing follow-up. Of these, 194,072 were under age 55 years at baseline and received a physical examination and a questionnaire about sociodemographic, lifestyle and health information, and provided blood samples^46^. Blood samples and other biospecimens were collected across assessment centers using standardized protocols and processed centrally with uniform quality control procedures^47,48^. Details on UK Biobank data collection are available online (www.ukbiobank.ac.uk).

#### Assessment of systemic aging

**Extended Data Fig. 5:**
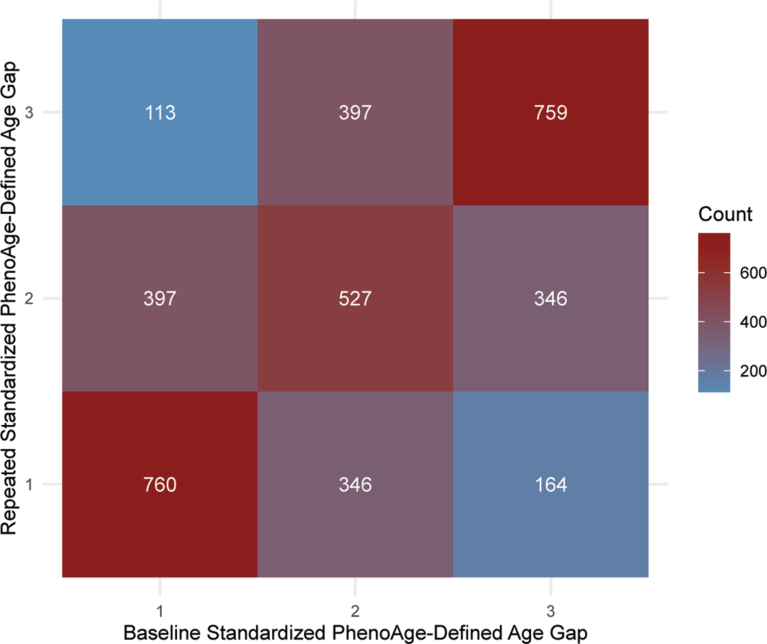
Concordance of standardized PhenoAge-defined age gap between baseline and repeated measurements. Among 3,809 participants with baseline and repeated blood draws (mean interval 4.4 years, s.d. 0.8), 60% of individuals in the lowest tertile and 60% in the highest tertile remained in the same tertile at follow-up.

PhenoAge^15^, validated across multi-ethnic cohorts^49^, is a mortality and morbidity trained composite measure of biological aging profile based on chronological age and nine blood biochemistry measurements (albumin, alkaline phosphatase, creatinine, C-reactive protein, glucose, mean cell volume, erythrocyte distribution width, leukocyte count and lymphocyte ratio). PhenoAge was derived in two steps. First, using data from 9,926 adults in National Health and Nutrition Examination Survey III, a Cox penalized regression model was applied to select a parsimonious set of nine measures plus chronological age from 42 candidate clinical measures that jointly predicted all-cause mortality. Second, these selected variables were entered into a parametric proportional hazards model assuming a Gompertz mortality distribution to estimate each participant’s 10-year mortality risk. Finally, this predicted risk was translated into units of years by identifying the chronological age in the reference population that corresponds to the same 10-year mortality risk. This equivalent age is defined as PhenoAge, representing the biological age implied by a person’s mortality risk profile rather than their actual years lived. Although trained on mortality, PhenoAge has been shown to correlate with morbidity^49^, and is therefore used here as a systemic indicator of biological aging and a mortality- and morbidity-based aging measure. In previous comparative analyses of blood chemistry-based aging measures, PhenoAge demonstrated stronger concordance with DNA methylation-based clocks than alternative clinical measurement-derived metrics, including the KDM and homeostatic dysregulation^50^. For example, correlations with the GrimAge methylation clock were higher for PhenoAge (*r* = 0.35) than for KDM (*r* = 0.25) or homeostatic dysregulation (*r* = 0.26)^50^. To assess the within-person variability of PhenoAge measurement, we leveraged repeated assessments available among 3,809 participants with baseline and repeat blood draws (mean interval 4.4 years, s.d. 0.8), 60% of participants in the lowest tertile and 60% in the highest tertile at baseline remained in the same tertile at follow-up (Extended Data Fig. 5).

KDM biological age^19^, in contrast, is trained to predict chronological age, integrating chronological age with eight biochemistry measurements (albumin, alkaline phosphatase, blood urea nitrogen, creatinine, C-reactive protein, glucose, HbA1c and total cholesterol) and two clinical measurements (systolic blood pressure and forced expiratory volume). KDM biological age was constructed using the KDM—a multivariate regression framework that estimates biological age as a weighted linear combination of measurements based on their individual associations with chronological age and their measurement variance.

In brief, both PhenoAge and KDM models were trained in the National Health and Nutrition Examination Survey III. In our analyses, we applied trained PhenoAge and KDM models to estimate biological age in the UK Biobank using the R package ‘BioAge.’ We further restricted the participants to those without missing or extreme values (>5 s.d.) for each measure. The level of age gap was determined by residuals from a linear regression of biological age against chronological age and was standardized and divided into tertiles. A total of 154,169 participants under age 55 years at baseline were included in the primary analyses.

#### Assessment of metabolomic aging

Metabolomic aging was estimated using a metabolomic aging score derived from nuclear magnetic resonance (NMR) metabolomics data generated by Nightingale Health for 249,616 UK Biobank participants^20^. In brief, out of 325 NMR metabolomic measurements, Zhang and colleagues^20^ performed a least absolute shrinkage and selection operator Cox regression model developed amongst 234,553 participants from England and Wales with all-cause mortality as the endpoint and selected 54 representative aging-related NMR measurements. A linear combination of 54 aging-related measurements weighted by the estimated coefficients from the model was used to produce an estimated metabolomic aging score for each participant. Metabolomic-based age gap was defined as the residual of metabolomic aging score regressed on chronological age, following an approach analogous to PhenoAge and KDM aging clocks. Higher values represent a metabolically older profile relative to a participant’s chronological age. A total of 140,373 participants under age of 55 years at baseline were included in the primary analyses.

#### Assessment of organ-specific aging

Organ-specific aging^14^ was estimated on 44,952 UK Biobank participants with plasma proteomic data available. Plasma proteomics profiling was performed using the Olink Explore 3072 platform, measuring 2,923 proteins across eight panels (cardiometabolic, cardiometabolic II, inflammation, inflammation II, neurology, neurology II, oncology and oncology II). Protein expression values were generated, normalized, and batch-corrected using the standardized UK Biobank preprocessing pipeline, including internal controls and quality control procedures, to minimize technical and center-related variation^48^. Organ-specific aging scores were calculated using previously published protein coefficients from Goeminne and colleagues^14^. In brief, Goeminne and colleagues analyzed plasma proteomic data from 53,014 UK Biobank participants and retained 44,952 participants and 2,916 proteins after filtering on missingness. Fivefold cross-validation with k-nearest neighbors imputation (*k* = 10) was used to train Cox proportional hazards elastic-net models to predict time-to-death, where elastic net linearly combines L1 and L2 penalties to allow simultaneous feature selection and shrinkage. Models were optimized to minimize the mean absolute error of the residuals, and detailed model performance metrics were reported in Supplementary Table 2 of ref. ^14^. Organ-enriched proteins were defined based on genotype-tissue expression data as those with expression levels at least fourfold higher in one organ compared with all others, and organ-specific clocks were trained using protein subsets corresponding to each organ^51^. Organ-specific aging was defined as the residual from an ordinary linear regression of the organ-specific predicted age (or predicted log mortality hazard) on chronological age, similar to PhenoAge. Each organ-specific aging score was estimated independently and analyzed in separate models rather than entered simultaneously into the same regression model. In addition to brain, pituitary, salivary, thyroid, esophagus, lung, heart, artery, liver, stomach, pancreas, kidney, intestine, adrenal, immune, skin, muscle and adipose aging, we included additional models for organismal aging (based on proteins expressed across several organs), multi-organ aging (based on proteins from all organs) and conventional aging (based on all available plasma proteins to predict chronological age). A total of 19,874 participants under age 55 years at baseline were included in this exploratory analysis.

#### Ascertainment of early-onset solid cancers

Incident cancer cases were identified through linkage to cancer registries and death records provided by the National Health Service (NHS) Information Centre and the NHS Central Register, National Records of Scotland, defined using the International Classification of Diseases, Tenth Revision (ICD-10) code. We included cancers of central nervous system (C70, C71, C72), head and neck (C00, C10-C14, C30–C32), thyroid (C73), lung (C33–C34), breast (C50), melanoma (C43), GI (C15–C26), colorectal (C18–C20), other GI (C15–C17, C21–C26), uterine (C54–C55), ovarian (C56) and prostate (C61). Complete cancer registry follow-up for all participants was available up to 31 December 2020 for England, 31 December 2016 for Wales and 30 November 2021 for Scotland. The mean (s.d.) follow-up was 6.4 (3.8) years.

Our primary outcome was incident early-onset solid cancers diagnosed between ages 18 and 55 years; later-onset cancers diagnosed after age 55 were examined secondarily. We selected 55 years as the primary cutoff for two reasons: (1) Consistency with recent epidemiologic evidence^2,3^, successive birth cohorts experiencing higher cancer incidence before age 50 seem to be carrying this excess risk into ages 50–54 years. Correspondingly, incidence rates among individuals younger than 55 have increased since the mid-1990s^2,3,52^. (2) The second reason was to maximize statistical power across cancer sites and enable analyses on subsites and organ-specific aging. Because measurements such as leukocyte count and lymphocyte ratio can vary substantially in hematological malignancies, we focused on solid tumors. For type-specific analyses, we restricted to cancer types with at least ten cases in each tertile of age gap. To assess the association between age gap and early-onset cancers, we excluded any cancer diagnosis (except nonmelanoma skin cancer) before or within 6 months of baseline, those who were underweight (body mass index (BMI) <18.5 kg m^−2^), or those with missing data on genetic ancestry.

#### Assessment of covariates

At baseline, participants self-reported age, sex, race, education, smoking status and pack-years, alcohol intake, 24-h recall and food frequency questionnaire (FFQ), family history of principal cancers (lung, breast, colorectal, prostate), age at menarche, oral contraceptive use, age at menopause or hysterectomy, parity and personal history of chronic obstructive pulmonary disease, cardiovascular disease (heart attack, stroke, heart failure, coronary heart disease, atrial fibrillation) and diabetes. Height and weight were measured to calculate BMI. The Townsend Deprivation Index was used to assess socioeconomic status with higher value indicating more deprivation. Total physical activity (metabolic equivalent of task) was measured using International Physical Activity Questionnaire. A healthy diet indicator was defined as meeting at least four of seven recommended dietary components for cardiometabolic health, based on available 24-h recall and FFQ data (fruit and vegetable intake, whole grains, red and processed meat, fish and alcohol intake)^53^. We also retrieved information on the first ten principal components (PCs) of genetic ancestry. Leukocyte telomere length was measured from peripheral blood at baseline using quantitative polymerase chain reaction.

#### Assessment of genetic predisposition of aging and cancer

To assess genetic predisposition to both aging and cancer, we calculated polygenic risk scores (PRS) derived from relevant single-nucleotide polymorphisms. Two sets of aging PRS were included: one for longevity^33^ and one for lifespan modified by nongenetic risk factors (that is, 13 diseases and 12 mortality risk factors)^34^. Cancer PRS were based on single-nucleotide polymorphisms identified in meta-analyses of genome-wide association studies of lung^35^, colorectal^36^ and endometrial^37^ cancers. For analyses that also adjusted for PRS, we restricted the analyses to 105,628 after excluding participants with low quality or abnormal heterozygosity, more than 5% missing heterozygosity, sex chromosome aneuploidy and a kinship coefficient greater than or equal to 0.0442 (ref. ^54^).

### All of Us Research Program

#### Study design and population

To validate our findings from the UK Biobank in EHR, we conducted an independent observational cohort study using the All of Us Research Program (Fig. 1a), a diverse US biomedical cohort of over 450,000 adults, with enrollment and follow-up ongoing since 2010. At enrollment, participants completed The Basics, Overall Health, Lifestyle and Personal and Family Health History surveys, consented to EHR retrieval, and provided physical measurements and biospecimen samples. We included 14,851 participants aged under 55 years at enrollment. We applied additional exclusions per the UK Biobank protocol. All participants provided written informed consent to share EHRs, surveys and other study data with qualified investigators for broad-based research.

#### Assessment of systemic aging

To estimate PhenoAge, we retrieved the nine measurements from each participant’s EHR before enrollment, selecting measurements that were closest in date to each other and recorded within a 5-year window, and calculated the mean chronological age between the first and the last measurement. We then applied the same methods as UK Biobank to estimate the degree of age gap. For the analyses of trends of age gap by birth cohort, a total of 10,262 participants were included.

#### Ascertainment of early-onset solid cancers

Incident solid cancer cases were identified through EHR after enrollment, defined using ICD-9-CM and ICD-10-CM codes in the All of Us Research Program release R2022Q4R9. All participants were followed-up until 1 July 2022. The mean (s.d.) follow-up was 2.4 (1.4) years. We assessed the association between age gap and early-onset cancers in 8,935 participants, excluding previous cancer diagnosis (except nonmelanoma skin cancer) before or within 6 months of the first PhenoAge measurement or who were underweight.

#### Assessment of covariates

At enrollment, participants self-reported age, sex, race/ethnicity, education, smoking status, alcohol intake status, personal history of chronic obstructive pulmonary disease, cardiovascular disease (heart attack, stroke, heart failure, coronary heart disease, atrial fibrillation) and diabetes, family history of lung, breast, colorectal or prostate cancer, and zip-code. The Social Deprivation Index—an area-level census-based measure using first three-digit zip-code—served as a proxy for socioeconomic status, with higher values indicating greater deprivation. BMI was obtained directly from the EHR or estimated using weight and height data closest to enrollment.

### Statistical analysis

A similar analytical approach was applied to the UK Biobank and the All of Us Research Program, if appropriate. In brief, we first assessed age gap levels by birth cohorts using linear regressions and evaluated sex differences by including an interaction term between birth year and sex, adjusted for age and age squared. Pairwise comparisons within age groups were conducted using two-tailed *t*-tests with Bonferroni-adjusted *P* values. Generalized additive models (GAMs) were used to visualize birth year trends in age gap, adjusted for age and age squared, separately by sex. Formal model comparison demonstrated better fit for the nonlinear specification than for linear models (*P*_likelihood ratio test_ < 0.001). To further examine differences in trends according to sex, we compared a model with sex-specific smooth terms to a model with a common smooth term using a likelihood ratio test, adjusting for age and age squared.

To assess the association between age gap and risk of early-onset cancers, multivariable Cox proportional hazards models were used to estimate hazard ratios (HRs) and 95% confidence intervals (CIs), using chronological age as the time scale. The proportional hazards assumption was evaluated using Schoenfeld residuals for each main model; no violations were detected (global test *P* values > 0.10). Age gap was analyzed by tertiles and as a continuous variable. To account for multiple comparisons across biological aging metrics and cancer outcomes, false discovery rate (FDR)-adjusted *P* values were calculated using the Benjamini–Hochberg procedure across all cancer site-specific tests presented in each table or figure. All models were adjusted for sex (male, female), race (White, other), Townsend Deprivation Index/Social Deprivation Index (continuous), education (pre-, postcollege), BMI (continuous), smoking status and intensity (never smoker, past smoker 1–19 pack-years, past smoker >19 pack-years, past smoker unknown pack-year, current smoker 1–19 pack-years, current smoker >19 pack-years, current smoker unknown pack-year), alcohol intake (never drinker, previous drinker, current drinker 0.1–14.9 g day^−1^, current drinker 15-29.9 g day^−1^, current drinker 30+ g day^−1^, current drinker but unknown g day^−1^), healthy diet (yes, no), physical activity (metabolic equivalent of task hours per week, in quartile), personal history of chronic obstructive pulmonary disease (yes, no), cardiovascular disease (yes, no) and diabetes (yes, no), and the first ten PCs of genetic ancestry (UK Biobank only). For cancer subtype analyses, we also adjusted for family history of lung, breast, colorectal and prostate cancer (yes, no), respectively. For female-specific cancers, we also adjusted for age at menarche (continuous), oral contraceptive use (never, past, current), menopause (yes, no, hysterectomy) and parity (continuous).

In the UK Biobank, we also assessed whether the association between age gap and early-onset cancers was independent of well-established markers of aging and cancer, by additionally adjusting for leukocyte telomere length, aging PRS^33,34^, and cancer-specific PRS (lung^35^, colorectal^36^ and endometrial^37^ cancers). For sensitivity analyses, we excluded participants with follow-up <2 years, and redefined early-onset cancers using age at diagnosis before 50 years. For secondary analysis, we assessed the association with late-onset cancers using similar approaches.

Associations between each organ-specific aging measures and early-onset solid cancers were assessed using Cox regression models using the same set of covariates as above, and FDR-adjusted *P* values were calculated using the Benjamini–Hochberg procedure. We also adjusted for PhenoAge-defined age gap.

For All of Us Research Program, we conducted stratified analyses for participants with (1) the nine measurements measured within 3 years, and also (2) the duration from first measurement to enrollment being less than 4 years. We also performed the sensitivity analysis excluding participants with less than 2 years of follow-up to minimize reverse causation.

This study followed the Strengthening the Reporting of Observational Studies in Epidemiology—Molecular Epidemiology (STROBE) reporting guideline. All analyses were performed using R (version 4.3.3) in RStudio (version 2026.01.2 + 418.pro1) with packages including tidyverse (v2.0.0), survival (v3.5-7), Publish (v2023.1.17), mgcv (v1.9-1), ggplot (v3.5.2) and BioAge (v0.1.0). All analyses were considered statistically significant with two-sided *P* < 0.05. Figures were generated in RStudio and subsequently refined for presentation using BioRender (https://www.biorender.com) and Adobe Illustrator 2026 (version 30.3).

### Ethics

The UK Biobank obtained ethical approval from the Northwest Multicenter Research Ethics Committee, the National Information Governance Board for Health and Social Care in England and Wales, and the Community Health Index Advisory Group in Scotland. All participants had provided written informed consent. UK Biobank received ethical approval from the Research Ethics Committee (REC reference 11/NW/0382). This study was conducted under UK Biobank Application No. 55288.

The All of Us Research Program obtained institutional review board (IRB) approval for all study procedures, and all participants provided informed consent at enrollment. To protect participant privacy, data used in this study were accessed by approved researchers only after registration in accordance with All of Us Research Program policies, completion of required ethics training, and agreement to data use terms through the All of Us Research Workbench (https://workbench.researchallofus.org/login).

This study used deidentified data from both resources. According to the Washington University School of Medicine in St. Louis Institutional Review Board, this study was deemed exempt from review as a secondary analysis of existing, deidentified data.

### Reporting summary

Further information on research design is available in the Nature Portfolio Reporting Summary linked to this article.

## Online content

Any methods, additional references, Nature Portfolio reporting summaries, source data, extended data, supplementary information, acknowledgements, peer review information; details of author contributions and competing interests; and statements of data and code availability are available at 10.1038/s41591-026-04448-w.

## Supplementary information

Supplementary Information Supplementary Tables 1–8.

Reporting Summary

## Data Availability

The data analyzed in this study were obtained from UK Biobank portal (https://www.ukbiobank.ac.uk/, email: ukbiobank@ukbiobank.ac.uk) and All of Us Research Program portal (https://www.researchallofus.org/, support@researchallofus.org), subject to controlled access. Researchers can apply to access UK Biobank data through the UK Biobank Access Management System (https://www.ukbiobank.ac.uk/enable-your-research/apply-for-access). Access to All of Us data is available via the Researcher Workbench (https://www.researchallofus.org/), following registration, training and data use agreement requirements. The genome-wide association studies summary statistics used for calculating PRS are available at https://www.ebi.ac.uk/gwas/, with contact email at gwas-info@ebi.ac.uk. The weights used for the PRS are available at PGS Catalog (https://www.pgscatalog.org/; pgs-info@ebi.ac.uk), at https://www.pgscatalog.org/publication/PGP000237/, https://www.pgscatalog.org/publication/PGP000095/, https://www.pgscatalog.org/publication/PGP000148/, https://www.pgscatalog.org/publication/PGP000491/ and https://www.pgscatalog.org/publication/PGP000344/.

## Extended data

**Extended Data Table 1:** Baseline characteristics of 148,317 participants under age 55 according to tertiles of standardized PhenoAge-defined age gap, UK Biobank (2006-2021)

	Standardized PhenoAge-Defined Age Gap (Tertiles)		
	T1 [Lowest]	T2	T3 [Highest]
**N**	**49,439**	**49,439**	**49,439**
Age at baseline, year, mean (s.d.)	48.2 (4.2)	48.2 (4.2)	48.2 (4.1)
PhenoAge, mean (s.d.)	31.7 (4.7)	36.4 (4.2)	42.4 (5.4)
Standardized PhenoAge-defined age gap, mean (s.d.)	−1.0 (0.4)	−0.07 (0.2)	1.1 (0.7)
Female, *n* (%)	29,379 (59%)	25,215 (51%)	25,669 (52%)
White, *n* (%)	46,030 (93%)	45,567 (92%)	44,070 (89%)
Townsend Deprivation Index, mean (s.d.)	−1.4 (3.0)	−1.2 (3.1)	−0.5 (3.4)
Post-college, *n* (%)	24,362 (49%)	21,949 (44%)	18,897 (38%)
BMI, kg/m^2^, mean (s.d.)	25.3 (3.6)	27.1 (4.2)	29.3 (5.7)
Smoking status, *n* (%)			
Never	31,948 (65%)	30,667 (62%)	26,917 (54%)
Past	13,875 (28%)	13,306 (27%)	12,085 (24%)
Current	3,616 (7.3%)	5,465 (11%)	10,436 (21%)
Pack-years among ever smokers, mean (s.d.)	14.6 (11.5)	17.8 (13.5)	22.4 (15.7)
Alcohol consumption, *n* (%)			
Never	1,668 (3.4%)	1,950 (3.9%)	2,580 (5.2%)
Past	1,295 (2.6%)	1,374 (2.8%)	2,022 (4.1%)
Current	46,476 (94%)	46,114 (93%)	44,836 (91%)
Per day in grams among current drinkers, mean (s.d.)	22.0 (18.6)	23.3 (21.5)	24.5 (25.8)
Healthy diet, *n* (%)			
Yes	25,268 (51%)	21,767 (44%)	18,179 (37%)
No	12,905 (26%)	15,468 (31%)	16,223 (33%)
Missing	11,266 (23%)	12,203 (25%)	15,036 (30%)
Family history of cancer, *n* (%)	15,365 (31%)	15,303 (31%)	15,325 (31%)
Lung cancer	4,591 (9.3%)	4,905 (9.9%)	5,292 (11%)
Breast cancer	5,092 (10%)	4,863 (9.8%)	4,698 (9.5%)
Colorectal cancer	4,444 (9%)	4,451 (9%)	4,449 (9%)
Prostate cancer	3,829 (7.7%)	3,754 (7.6%)	3,560 (7.2%)

Baseline characteristics of 148,317 participants aged <55 years in the UK Biobank (2006–2021), stratified by tertiles of standardized PhenoAge-defined age gap (T1: lowest; T3: highest). Continuous variables are presented as mean (s.d.), and categorical variables are presented as number (percentage). Healthy diet was defined as meeting at least four of seven recommended dietary components for cardiometabolic health, based on available 24-h recall and FFQ data (fruit and vegetable intake, whole grains, red and processed meat, fish and moderate alcohol intake).

Abbreviations: BMI, body mass index; FFQ, Food Frequency Questionnaire; s.d., standard deviation.

**Extended Data Table 2:** Associations between PhenoAge-defined age gap and risk of early-onset solid cancers, UK Biobank (2006-2021)

Cancer Sites	Standardized PhenoAge-Defined Age Gap (Tertiles)			Per 1 s.d. Increase	P_trend_^h^
	T1 [Lowest]	T2	T3 [Highest]		
**All Solid Cancer**					
No_case_	951	954	1079		
Unadjusted HR (95%CI)	1 [Ref]	1.00 (0.92 to 1.10)	1.15 (1.05 to 1.26)	1.08 (1.04 to 1.12)	4.7×10^−5^
Adjusted HR (95%CI)^a,b,c,e,f^	1 [Ref]	1.05 (0.95 to 1.17)	1.15 (1.03 to 1.28)	1.08 (1.03 to 1.13)	0.002
**Central Nervous System**					
No_case_	25	20	18		
Unadjusted HR (95%CI)	1 [Ref]	0.79 (0.44 to 1.43)	0.72 (0.39 to 1.31)	0.87 (0.67 to 1.12)	0.28
Adjusted HR (95%CI)^a^	1 [Ref]	0.89 (0.44 to 1.79)	0.67 (0.29 to 1.57)	0.98 (0.69 to 1.40)	0.92
**Head and Neck**					
No_case_	34	35	36		
Unadjusted HR (95%CI)	1 [Ref]	1.03 (0.64 to 1.65)	1.08 (0.67 to 1.73)	1.09 (0.91 to 1.32)	0.35
Adjusted HR (95%CI)^a^	1 [Ref]	0.84 (0.47 to 1.49)	0.81 (0.43 to 1.52)	1.00 (0.77 to 1.31)	0.98
**Thyroid**					
No_case_	27	14	20		
Unadjusted HR (95%CI)	1 [Ref]	0.51 (0.27 to 0.97)	0.72 (0.40 to 1.29)	0.91 (0.70 to 1.17)	0.46
Adjusted HR (95%CI)^a^	1 [Ref]	0.44 (0.20 to 0.97)	0.62 (0.28 to 1.34)	0.82 (0.57 to 1.16)	0.26
**Lung**					
No_case_	16	28	58		
Unadjusted HR (95%CI)	1 [Ref]	1.75 (0.95 to 3.24)	3.70 (2.12 to 6.45)	1.83 (1.56 to 2.15)	1.5×10^−13^
Adjusted HR (95%CI)^a,b^	1 [Ref]	1.60 (0.73 to 3.49)	2.34 (1.11 to 4.96)	1.57 (1.24 to 1.97)	1.3×10^−4^
**Breast**					
No_case_	452	405	401		
Unadjusted HR (95%CI)	1 [Ref]	1.02 (0.90 to 1.17)	1.00 (0.87 to 1.14)	1.00 (0.95 to 1.05)	0.94
Adjusted HR (95%CI)^a,c,d^	1 [Ref]	1.08 (0.93 to 1.26)	1.05 (0.88 to 1.24)	1.01 (0.94 to 1.08)	0.79
**Melanoma**					
No_case_	97	93	90		
Unadjusted HR (95%CI)	1 [Ref]	0.96 (0.72 to 1.28)	0.97 (0.72 to 1.29)	0.98 (0.87 to 1.10)	0.73
Adjusted HR (95%CI)^a^	1 [Ref]	0.96 (0.70 to 1.32)	0.93 (0.65 to 1.32)	0.98 (0.84 to 1.14)	0.79
**Gastrointestinal**					
No_case_	111	141	187		
Unadjusted HR (95%CI)	1 [Ref]	1.26 (0.99 to 1.62)	1.69 (1.34 to 2.15)	1.23 (1.13 to 1.35)	5.1×10^−6^
Adjusted HR (95%CI)^a,e^	1 [Ref]	1.21 (0.94 to 1.56)	1.52 (1.18 to 1.96)	1.17 (1.06 to 1.30)	0.002
**Colorectal**					
No_case_	84	96	113		
Unadjusted HR (95%CI)	1 [Ref]	1.13 (0.85 to 1.52)	1.34 (1.01 to 1.78)	1.13 (1.01 to 1.26)	0.04
Adjusted HR (95%CI)^a,e^	1 [Ref]	1.14 (0.85 to 1.54)	1.37 (1.01 to 1.86)	1.14 (1.01 to 1.29)	0.04
**Other Gastrointestinal Cancers**					
No_case_	27	45	74		
Unadjusted HR (95%CI)	1 [Ref]	1.67 (1.03 to 2.69)	2.80 (1.80 to 4.35)	1.45 (1.25 to 1.68)	8.2×10^−7^
Adjusted HR (95%CI)^a^	1 [Ref]	1.36 (0.75 to 2.48)	2.10 (1.17 to 3.77)	1.25 (1.01 to 1.55)	0.04
**Uterine**					
No_case_	26	24	51		
Unadjusted HR (95%CI)	1 [Ref]	1.07 (0.62 to 1.87)	2.26 (1.40 to 3.64)	1.45 (1.22 to 1.72)	2.1×10^−5^
Adjusted HR (95%CI)^a,d^	1 [Ref]	0.94 (0.47 to 1.85)	1.86 (0.99 to 3.48)	1.31 (1.04 to 1.66)	0.02
**Ovarian**					
No_case_	30	25	36		
Unadjusted HR (95%CI)	1 [Ref]	0.82 (0.50 to 1.34)	1.06 (0.67 to 1.68)	1.05 (0.87 to 1.26)	0.64
Adjusted HR (95%CI)^a,d^	1 [Ref]	1.24 (0.66 to 2.30)	1.58 (0.83 to 3.00)	1.26 (0.98 to 1.63)	0.07
**Prostate**					
No_case_	65	82	79		
Unadjusted HR (95%CI)	1 [Ref]	1.04 (0.75 to 1.44)	1.04 (0.75 to 1.44)	1.00 (0.87 to 1.15)	0.96
Adjusted HR (95%CI)^a,f,g^	1 [Ref]	1.06 (0.72 to 1.56)	1.18 (0.79 to 1.79)	1.08 (0.90 to 1.29)	0.41

HRs and 95% CIs for the association between standardized PhenoAge-defined age gap and risk of early-onset solid cancers in the UK Biobank (2006–2021), estimated using Cox proportional hazards models. Age gap was analyzed both as tertiles (T1: lowest [reference], T2, T3: highest) and as a continuous variable per 1 s.d. increase. The number of incident cases in each tertile is provided.

^a^Adjusted for sex, race, the Townsend Deprivation Index, education, BMI, smoking status and intensity, alcohol intake per day, diet, physical activity, personal history of chronic obstructive pulmonary disease, cardiovascular disease, and diabetes, and the first 10 PCs of genetic ancestry.

^b^Additionally adjusted for family history of lung cancer.

^c^Additionally adjusted for family history of breast cancer.

^d^ Calculated among female participants only and additionally adjusted for age at menarche, oral contraceptive use, menopause, and parity.

^e^Additionally adjusted for family history of colorectal cancer.

^f^Additionally adjusted for family history of prostate cancer.

^g^Calculated among male participants only.

^h^P_trend_ was calculated using standardized PhenoAge-defined age gap as a continuous variable.

Abbreviations: BMI, body mass index; CI, confidence interval; HR, hazard ratio; PC, principal component; s.d., standard deviation.

**Extended Data Table 3:** Associations between PhenoAge-defined age gap and risk of late-onset lung, gastrointestinal, and uterine cancer, UK Biobank (2006-2021)

Cancer Sites	Standardized PhenoAge-Defined Age Gap (Tertiles)			Per 1 s.d. Increase	P_trend_^e^	P_trend-FDR_^f^
	T1 [Lowest]	T2	T3 [Highest]			
**Lung** ^**a**^						
No_case_	58	64	138			
Unadjusted HR (95%CI)	1 [Ref]	1.11 (0.78 to 1.59)	2.43 (1.78 to 3.30)	1.48 (1.33 to 1.65)	8.6×10^−13^	4.3×10^−12^
Adjusted HR (95%CI)^b^	1 [Ref]	1.10 (0.71 to 1.71)	1.49 (0.98 to 2.27)	1.10 (0.94 to 1.29)	0.23	0.25
**Gastrointestinal**						
No_case_	266	309	402			
Unadjusted HR (95%CI)	1 [Ref]	1.18 (1.00 to 1.39)	1.57 (1.35 to 1.84)	1.24 (1.17 to 1.31)	8.1×10^−14^	8.1×10^−13^
Adjusted HR (95%CI)^b,c^	1 [Ref]	1.06 (0.90 to 1.25)	1.24 (1.05 to 1.47)	1.12 (1.05 to 1.20)	0.001	0.002
**Colorectal**						
No_case_	178	195	222			
Unadjusted HR (95%CI)	1 [Ref]	1.11 (0.90 to 1.35)	1.29 (1.05 to 1.57)	1.14 (1.05 to 1.23)	0.002	0.002
Adjusted HR (95%CI)^b,c^	1 [Ref]	1.02 (0.83 to 1.26)	1.11 (0.89 to 1.38)	1.07 (0.98 to 1.17)	0.14	0.18
**Other Gastrointestinal Cancers**						
No_case_	88	114	180			
Unadjusted HR (95%CI)	1 [Ref]	1.32 (1.00 to 1.75)	2.16 (1.67 to 2.79)	1.40 (1.28 to 1.54)	1.4×10^−12^	4.67×10^−12^
Adjusted HR (95%CI)^b^	1 [Ref]	1.17 (0.83 to 1.64)	1.51 (1.07 to 2.13)	1.25 (1.09 to 1.43)	0.002	0.003
**Uterine**						
No_case_	51	50	82			
Unadjusted HR (95%CI)	1 [Ref]	1.16 (0.78 to 1.71)	1.89 (1.33 to 2.69)	1.35 (1.19 to 1.54)	5.2×10^−6^	1.3×10^−5^
Adjusted HR (95%CI)^b,d^	1 [Ref]	0.86 (0.54 to 1.36)	1.30 (0.82 to 2.04)	1.05 (0.88 to 1.26)	0.58	0.58

HRs and 95% CIs for the association between standardized PhenoAge-defined age gap and risk of selected late-onset cancers (lung, gastrointestinal, and uterine) in the UK Biobank (2006–2021), estimated using Cox proportional hazards models. Age gap was analyzed as tertiles (T1: lowest [reference], T2, T3: highest) and per 1 s.d. increase. The number of incident cases in each tertile is provided.

^a^Additionally adjusted for family history of lung cancer.

^b^Adjusted for sex, race, the Townsend Deprivation Index, education, BMI, smoking status and intensity, alcohol intake per day, diet, physical activity, personal history of chronic obstructive pulmonary disease, cardiovascular disease, and diabetes, and the first 10 PCs of genetic ancestry.

^c^Additionally adjusted for family history of colorectal cancer.

^d^Calculated among female participants only and additionally adjusted for age at menarche, oral contraceptive use, menopause, and parity.

^e^P_trend_ was calculated using standardized PhenoAge-defined age gap as a continuous variable.

^f^P_trend-FDR_ was calculated across the five cancer site-specific tests presented in this table.

Abbreviations: BMI, body mass index; CI, confidence interval; HR, hazard ratio; PC, principal component, s.d., standard deviation.

**Extended Data Table 4:** Baseline characteristics of 8,935 participants under age 55 according to tertiles of standardized PhenoAge-defined age gap, All of Us Research Program (2010-2022)

N	Standardized PhenoAge-Defined Age Gap (Tertiles)		
	T1 [Lowest]	T2	T3 [Highest]
	2,979	2,978	2,978
Age at enrollment, year, mean (s.d.)	41.4 (9.8)	39.7 (10.1)	40.3 (9.9)
Mean age between first and last measurement, year, mean (s.d.)	40.0 (9.9)	38.5 (10.2)	39.4 (9.9)
Years between first and last measurement, mean (s.d.)	1.4 (1.5)	1.3 (1.4)	1.0 (1.3)
Years between first measurement and enrollment, mean (s.d.)	2.1 (2.0)	1.9 (1.9)	1.3 (1.7)
PhenoAge, mean (s.d.)	34.4 (13.0)	40.8 (11.2)	55.3 (15.3)
Age gap, year, mean (s.d.)	−9.5 (7.7)	−1.4 (2.1)	12.0 (10.2)
Female, *n* (%)	2,301 (77%)	2,010 (68%)	1,708 (57%)
NHW, n (%)	1,728 (58%)	1,608 (54%)	1,393 (47%)
NHB, n (%)	368 (12%)	501 (17%)	585 (20%)
Hispanic, *n* (%)	667 (22%)	699 (24%)	856 (29%)
Social Deprivation Index, mean (s.d.)	0.3 (0.1)	0.3 (0.1)	0.3 (0.1)
Education level, *n* (%)			
<High school	198 (6.6%)	272 (9.1%)	403 (14%)
High school or GED certification	471 (16%)	740 (25%)	958 (32%)
Some college	935 (31%)	1,067 (36%)	1,038 (35%)
College	1,375 (46%)	899 (30%)	579 (19%)
BMI, kg/m^2^, mean (s.d.)	29.4 (7.0)	32.8 (8.9)	33.9 (10.8)
Smoking status, *n* (%)			
Never	2,169 (73%)	1,967 (66%)	1,834 (62%)
Past	584 (20%)	546 (18%)	581 (20%)
Current	226 (7.6%)	465 (16%)	563 (19%)
Alcohol consumption, *n* (%)			
Never	300 (10%)	352 (12%)	423 (14%)
Past	395 (13%)	491 (17%)	657 (22%)
Current	2,284 (77%)	2,135 (72%)	1,898 (64%)
Family history of cancer, *n* (%)	585 (20%)	449 (15%)	343 (12%)
Personal history of chronic illness, *n* (%)	216 (7.3%)	318 (11%)	370 (12%)
Chronic obstructive pulmonary disease	53 (1.8%)	65 (2.2%)	64 (2.1%)
Cardiovascular disease	104 (3.5%)	124 (4.2%)	158 (5.3%)
Diabetes	88 (3.0%)	180 (6.0%)	251 (8.4%)

Baseline characteristics of 8,935 participants aged <55 years in the All of Us Research Program (2010–2022), stratified by tertiles of standardized PhenoAge-defined age gap (T1: lowest; T3: highest). Continuous variables are presented as mean (s.d.), and categorical variables are presented as number (percentage). Abbreviations: BMI, body mass index; NHB, non-Hispanic Black; NHW, non-Hispanic White; s.d., standard deviation.

## Code availability

All code used for data processing, statistical analyses and model development for the UK Biobank analyses is publicly available on GitHub https://github.com/caolabwusm/EOCancerRiskAging. The repository includes scripts for data preprocessing, model implementation and reproduction of the main results. Code specific to the All of Us Research Program cannot be shared due to platform access and data use restrictions; however, detailed methodological descriptions are provided to enable reproducibility within the authorized research environment.
