# Supplementary material for: Biological aging and generational shifts in early-onset cancer risk
Source: Nat Med. 2026 Jun 22;32(8):2983–90. doi: 10.1038/s41591-026-04448-w (PMC13472952; doi:10.1038/s41591-026-04448-w)
Supplement: Supplementary file 1 [file 41591_2026_4448_MOESM1_ESM.pdf]

nature medicine

Supplementary information https://doi.org/10.1038/s41591-026-04448-w

Biological aging and generational shifts in early-onset cancer risk

In the format provided by the authors and unedited

Supplementary Online Content

Supplementary Table 1. Summary of birth year contribution in generalized additive model, UK Biobank (2006-2021) and All of Us Research Program (2010-2022).

Biobank	Model	$P_{Birth\ Cohort}$[a]	R^2	P_{LRT}[b]
**UK Biobank**	Overall	$<2.2\times10^{-16}$	0.003	$<2.2\times10^{-16}$
	Female	$<2.2\times10^{-16}$	0.007	$<2.2\times10^{-16}$
	Male	$<2.2\times10^{-16}$	0.005	2.1×10^{-11}
**All of Us Research Program**	Overall	$<2.2\times10^{-16}$	0.009	$<2.2\times10^{-16}$
	Female	$<2.2\times10^{-16}$	0.005	$<2.2\times10^{-16}$
	Male	$<2.2\times10^{-16}$	0.018	$<2.2\times10^{-16}$

a. P value for the contribution of birth cohort in the generalized additive model, assessing its association with age gap while adjusting for age and age squared.
b. P value from the likelihood ratio test (LRT), comparing the full model with birth cohort included to the reduced model without birth cohort, to evaluate the statistical significance of birth year contribution.

Supplementary Table 2. Sensitivity analysis of PhenoAge-defined age gap and risk of early-onset solid cancer using a <50-year age cutoff, UK Biobank (2006-2021).

Cancer Sites	Standardized PhenoAge-Defined Age Gap (Per 1 s.d. Increase)	P_{trend}[g]	$P_{trend\text{-}FDR}$[h]
**All Solid Cancer**			
N_{case}	954		
Adjusted HR (95%CI)[a,b,c,e,f]	1.12 (1.03 to 1.22)	0.005	0.025
**Lung**			
N_{case}	24		
Adjusted HR (95%CI)[a,b]	1.34 (0.91 to 1.96)	0.13	0.16
**Gastrointestinal**			
N_{case}	108		
Adjusted HR (95%CI)[a,e]	1.16 (0.94 to 1.42)	0.16	0.16
**Colorectal**			
N_{case}	75		
Adjusted HR (95%CI)[a,e]	1.24 (0.97 to 1.57)	0.09	0.15
**Uterine**			
N_{case}	23		
Adjusted HR (95%CI)[a,d]	1.75 (1.03 to 2.97)	0.04	0.10

a. Adjusted for sex, race, the Townsend Deprivation Index, education, BMI, smoking status and intensity, alcohol intake per day, diet, physical activity, personal history of chronic obstructive pulmonary disease, cardiovascular disease, and diabetes, and the first 10 PCs of genetic ancestry.

b. Additionally adjusted for family history of lung cancer.

c. Additionally adjusted for family history of breast cancer.

d. Calculated among female participants only and additionally adjusted for age at menarche, oral contraceptive use, menopause, and parity.

e. Additionally adjusted for family history of colorectal cancer.

f. Additionally adjusted for family history of prostate cancer.

g. P_{trend} was calculated using standardized PhenoAge-defined age gap as a continuous variable.

h. $P_{trend\text{-}FDR}$ was calculated using the Benjamini-Hochberg false discovery rate correction across the five cancer site–specific tests presented in this table.

Abbreviations: BMI, body mass index; CI, confidence interval; HR, hazard ratio; PC, principal component; s.d., standard deviation.

Supplementary Table 3. Mediation analysis on the association between PhenoAge-defined age gap and early-onset solid cancers, UK Biobank (2006-2021).

Cancer Sites	Adiposity<br>Per 5 kg/m² BMI	Physical Activity<br>Per 5 MET-hr/week	Alcohol<br>Per 10 g/d	Smoking<br>Per 5 pack-years
**All Solid Cancer**	1.03 (0.99 to 1.08)	0.99 (0.99 to 1.00)	1.03 (1.01 to 1.05)	1.05 (1.01 to 1.09)
**PNDE**[a]	0.98 (0.96 to 1.09)	0.99 (0.99 to 1.00)	1.01 (1.00 to 1.01)	1.01 (0.99 to 1.02)
**PNIE**[b]	1.00 (0.97 to 1.09)	0.99 (0.98 to 1.00)	0.99 (0.98 to 1.00)	1.00 (1.00 to 1.01)
**% Mediated by Age Gap**[c]	-9.2%	2.5%	-4.0%	23.6%

a. PNDE (Direct effect): Hazard ratio for the association between the lifestyle factor and cancer risk not operating through PhenoAge-defined age gap, holding PhenoAge-defined age gap constant.

b. PNIE (Indirect effect): Hazard ratio for the association between the lifestyle factor and cancer risk operating through PhenoAge-defined age gap.

c. % Mediated by PhenoAge-defined age gap: Proportion of the total association between the lifestyle factor and cancer risk explained by PhenoAge-defined age gap.

d. All models were adjusted for age, sex, race, socioeconomic index, education, BMI, physical activity, smoking, alcohol intake, prior cancer history, and first 10 PCs of genetic ancestry. The exposure of interest was excluded from the covariate set in both the outcome and mediator models to avoid collinearity.

Abbreviations: BMI, body mass index; MET, metabolic equivalent of task; PC, principal component; PNDE, Pure natural direct effect; PNIE, Pure natural indirect effect.

Supplementary Table 4. Baseline characteristics of 137,553 participants under age 55 according to tertiles of standardized KDM-defined age gap, UK Biobank (2006-2021).

	**Standardized KDM-Defined Age Gap (Tertiles)**		
	**T1 [Lowest]**	**T2**	**T3 [Highest]**
N	**45,851**	**45,851**	**45,851**
Age at baseline, year, mean (s.d.)	48.1 (4.10)	48.4 (4.16)	48.1 (4.22)
KDM, mean (s.d.)	69.6 (4.26)	77.9 (3.19)	86.4 (4.95)
Standardized KDM-defined age gap, mean (s.d.)	-1.08 (0.45)	-0.02 (0.26)	1.12 (0.56)
Female, *n* (%)	40,742 (89%)	24,445 (53%)	10,072 (22%)
White, *n* (%)	42,400 (92%)	42,176 (92%)	41,886 (91%)
Townsend Deprivation Index, mean (s.d.)	-1.16 (3.12)	-1.11 (3.17)	-0.94 (3.22)
Post-college, *n* (%)	21,849 (48%)	20,202 (44%)	18,651 (41%)
BMI, kg/m2, mean (s.d.)	25.2 (4.16)	27.2 (4.62)	28.9 (4.81)
Smoking status, *n* (%)			
Never	28,465 (62%)	28,140 (61%)	26,989 (59%)
Past	12,274 (27%)	11,842 (26%)	12,272 (27%)
Current	5,112 (11%)	5,868 (13%)	6,590 (14%)
Pack-years among ever smokers, mean (s.d.)	16.0 (12.4)	18.5 (14.0)	20.9 (15.2)
Alcohol consumption, *n* (%)			
Never	2,026 (4.4%)	1,895 (4.1%)	1,682 (3.7%)
Past	1,459 (3.2%)	1,395 (3%)	1,308 (2.9%)
Current	42,366 (92%)	42,560 (93%)	42,861 (93%)
Per day in grams among current drinkers, mean (s.d.)	18.4 (16.6)	22.6 (21.1)	28.0 (25.9)
Healthy diet, *n* (%)			
Yes	23,638 (52%)	20,659 (45%)	16,705 (36%)
No	10,814 (24%)	13,960 (30%)	16,566 (36%)
Missing	11,399 (24.9%)	11,231 (24.5%)	12,580 (27.4%)
Family history of cancer, *n* (%)	15,365 (31%)	15,303 (31%)	15,325 (31%)
Lung cancer	4,325 (9.4%)	4,646 (10%)	4,614 (10%)
Breast cancer	4,829 (11%)	4,620 (10%)	4,261 (9.3%)
Colorectal cancer	4,022 (8.8%)	4,249 (9.3%)	4,064 (8.9%)
Prostate cancer	3,590 (7.8%)	3,499 (7.6%)	3,252 (7.1%)

Abbreviations: BMI, body mass index; KDM, Klemera-Doubal method; s.d., standard deviation.

Supplementary Table 5. Associations between KDM-defined age gap and risk of early-onset solid cancers, UK Biobank (2006-2021).

Cancer Sites	Standardized KDM-Defined Age Gap (Tertiles)			Per 1 s.d. Increase	P_{trend}[g]
	T1 [Lowest]	T2	T3 [Highest]		
**All Solid Cancer**					
N_{case}	1,198	887	719		
Adjusted HR (95%CI)[a,b,c,e,f]	1 [Ref]	0.98 (0.88 to 1.09)	1.01 (0.88 to 1.16)	1.03 (0.97 to 1.09)	0.33
**Lung**					
N_{case}	28	31	30		
Adjusted HR (95%CI)[a,b]	1 [Ref]	1.53 (0.72 to 3.25)	2.74 (1.21 to 6.19)	1.53 (1.11 to 2.11)	0.01
**Gastrointestinal**					
N_{case}	118	126	172		
Adjusted HR (95%CI)[a,e]	1 [Ref]	1.37 (0.99 to 1.90)	1.70 (1.17 to 2.48)	1.41 (1.22 to 1.64)	5.6×10^{-6}
**Colorectal**					
N_{case}	84	83	116		
Adjusted HR (95%CI)[a,e]	1 [Ref]	1.30 (0.88 to 1.92)	1.83 (1.18 to 2.85)	1.49 (1.25 to 1.77)	6.8×10^{-6}
**Uterine**					
N_{case}	47	31	13		
Adjusted HR (95%CI)[a,d]	1 [Ref]	1.25 (0.71 to 2.19)	1.25 (0.57 to 2.73)	1.13 (0.81 to 1.57)	0.48

a. Adjusted for sex, race, the Townsend Deprivation Index, education, BMI, smoking status and intensity, alcohol intake per day, physical activity, personal history of chronic obstructive pulmonary disease, cardiovascular disease, and diabetes, and the first 10 PCs of genetic ancestry.

b. Additionally adjusted for family history of lung cancer.

c. Additionally adjusted for family history of breast cancer.

d. Calculated among female participants only and additionally adjusted for age at menarche, oral contraceptive use, menopause, and parity.

e. Additionally adjusted for family history of colorectal cancer.

f. Additionally adjusted for family history of prostate cancer.

g. P_{trend} was calculated using standardized KDM-defined age gap as a continuous variable.

Abbreviations: BMI, body mass index; CI, confidence interval; HR, hazard ratio; KDM, Klemera-Doubal method; PC, principal component; s.d., standard deviation.

Supplementary Table 6. Baseline characteristics of 140,373 participants under age 55 according to tertiles of standardized metabolomic-based age gap, UK Biobank (2006-2021).

	**Standardized Metabolomic-Based Age Gap (Tertiles)**		
	**T1 [Lowest]**	**T2**	**T3 [Highest]**
**N**	**46,791**	**46,791**	**46,791**
Age at baseline, year, mean (s.d.)	47.9 (4.17)	48.2 (4.17)	48.5 (4.14)
Metabolomic-based age gap, mean (s.d.)	-0.218 (0.415)	-0.0746 (0.413)	0.230 (0.462)
Female, *n* (%)	25,733 (55%)	24,327 (52%)	26,048 (56%)
White, *n* (%)	42,404 (91%)	43,120 (92%)	42,866 (92%)
Townsend Deprivation Index, mean (s.d.)	-1.16 (3.15)	-1.11 (3.16)	-0.831 (3.28)
Post-college, *n* (%)	22,066 (47%)	20,875 (45%)	18,994 (41%)
BMI, kg/m^2, mean (s.d.)	26.1 (4.13)	27.1 (4.56)	28.4 (5.48)
Smoking status, *n* (%)			
Never	29,051 (62%)	28,750 (61%)	27,290 (58%)
Past	13,089 (28%)	12,412 (26%)	11,577 (25%)
Current	4,705 (10%)	5,697 (12%)	7,802 (17%)
Pack-years among ever smokers, mean (s.d.)	4.49 (9.83)	5.31 (11.1)	7.17 (13.5)
Alcohol consumption, *n* (%)			
Never	1,542 (3.3%)	1,896 (4%)	2,426 (5.2%)
Past	1,046 (2.2%)	1,405 (3%)	2,003 (4.3%)
Current	44,257 (94%)	43,558 (93%)	42,240 (91%)
Per day in grams among current drinkers, mean (s.d.)	24.6 (21.2)	22.4 (20.7)	21.5 (21.9)
Healthy diet, *n* (%)			
Yes	21,110 (45%)	20,915 (45%)	19,926 (43%)
No	13,217 (28%)	14,248 (30%)	14,762 (32%)
Missing	12,518 (26.7%)	11,696 (25.0%)	11,981 (25.7%)
Family history of cancer, *n* (%)	11,115 (24%)	11,240 (24%)	10,963 (23%)
Lung cancer	4,430 (9.5%)	4,688 (10%)	4,857 (10%)
Breast cancer	4,632 (9.9%)	4,711 (10%)	4,556 (9.8%)
Colorectal cancer	4,134 (8.8%)	4,227 (9%)	4,241 (9.1%)
Prostate cancer	3,588 (7.7%)	3,561 (7.6%)	3,375 (7.2%)

Abbreviations: BMI, body mass index; s.d., standard deviation.

Supplementary Table 7. Associations between metabolomic-based age gap and early-onset cancers, UK Biobank (2006-2021).

Cancer Sites	Standardized Metabolomic-Based Age Gap (Tertiles)			Per 1 s.d. Increase	P_{trend}[g]
	T1 [Lowest]	T2	T3 [Highest]		
**All Solid Cancer**					
N_{case}	924	954	933		
Adjusted HR (95%CI)[a,b,c,d,e,f]	1 [Ref]	1.11 (0.99 to 1.23)	1.08 (0.96 to 1.20)	1.04 (0.99 to 1.08)	0.13
**Lung**					
N_{case}	13	25	56		
Adjusted HR (95%CI)[a,b]	1 [Ref]	1.97 (1.01 to 3.86)	4.61 (2.52 to 8.45)	1.89 (1.55 to 2.30)	2.5×10^{-10}
**Gastrointestinal**					
N_{case}	132	133	144		
Adjusted HR (95%CI)[a,e]	1 [Ref]	0.99 (0.77 to 1.26)	1.04 (0.82 to 1.33)	1.05 (0.95 to 1.16)	0.33
**Colorectal**					
N_{case}	87	90	94		
Adjusted HR (95%CI)[a,e]	1 [Ref]	1.05 (0.78 to 1.41)	1.13 (0.84 to 1.52)	1.09 (0.96 to 1.23)	0.19
**Uterine**					
N_{case}	21	31	43		
Adjusted HR (95%CI)[a,d]	1 [Ref]	1.52 (0.73 to 3.14)	2.43 (1.23 to 4.82)	1.44 (1.12 to 1.85)	0.005

a. Adjusted for sex, race, the Townsend Deprivation Index, education, BMI, smoking status and intensity, alcohol intake per day, physical activity, personal history of chronic obstructive pulmonary disease, cardiovascular disease, and diabetes, and the first 10 PCs of genetic ancestry.

b. Additionally adjusted for family history of lung cancer.

c. Additionally adjusted for family history of breast cancer.

d. Calculated among female participants only and additionally adjusted for age at menarche, oral contraceptive use, menopause, and parity.

e. Additionally adjusted for family history of colorectal cancer.

f. Additionally adjusted for family history of prostate cancer.

g. P_{trend} was calculated using standardized metabolomic-based age gap as a continuous variable.

Abbreviations: BMI, body mass index; CI, confidence interval; HR, hazard ratio; PC, principal component; s.d., standard deviation.

Supplementary Table 8. Associations between proteomic-based organ-specific aging and early-onset lung, gastrointestinal, and colorectal cancers, additionally adjusted for systemic aging (PhenoAge-defined age gap), UK Biobank (2006-2021).

Cancer Sites	Organ-specific Aging (Per 1 s.d. Increase)			Systemic Aging (Per 1 s.d. Increase)		
	**HR (95%CI)**	P_{trend}[d]	$P_{trend\text{-}FDR}$[e]	**HR (95%CI)**	P_{trend}[d]	$P_{trend\text{-}FDR}$[e]
**Lung**[a,b]						
NN_{case}	21			21		
Model 1: immune aging only	1.89 (1.20 to 2.97)	0.006	0.03	-	-	
Model 2: added systemic aging (PhenoAge)	1.85 (1.17 to 2.93)	0.008	0.03	1.23 (0.78 to 1.93)	0.37	0.37
**Gastrointestinal**[a,c]						
N_{case}	55			55		
Model 1: adipose aging only	1.36 (0.99 to 1.86)	0.06	0.07	-	-	
Model 2: added systemic aging (PhenoAge)	1.35 (0.99 to 1.85)	0.06	0.07	1.37 (1.00 to 1.87)	0.05	0.07
**Colorectal**[a,c]						
N_{case}	37			37		
Model 1: adipose aging only	1.60 (1.11 to 2.32)	0.01	0.03	-	-	
Model 2: added systemic aging (PhenoAge)	1.60 (1.11 to 2.32)	0.01	0.03	1.42 (0.98 to 2.08)	0.07	0.10

a. Adjusted for sex, race, the Townsend Deprivation Index, education, BMI, smoking status and intensity, alcohol intake per day, physical activity, personal history of chronic obstructive pulmonary disease, cardiovascular disease, and diabetes, and the first 10 PCs of genetic ancestry.

b. Additionally adjusted for family history of lung cancer.

c. Additionally adjusted for family history of colorectal cancer.

d. P_{trend} was calculated using standardized (organ-specific or PhenoAge-defined) age gap as a continuous variable and adjusted for multiple comparisons using FDR across all organ-specific and systemic aging models and cancer sites tested.

e. $P_{trend\text{-}FDR}$ was calculated using the Benjamini–Hochberg false discovery rate correction across the three cancer site-specific tests presented in this table.

Abbreviations: BMI, body mass index; CI, confidence interval; HR, hazard ratio; PC, principal component; s.d., standard deviation.
