# Supplementary material for: Biological aging and generational shifts in early-onset cancer risk
Source: Nat Med. 2026 Jun 22;32(8):2983–90. doi: 10.1038/s41591-026-04448-w (PMC13472952; doi:10.1038/s41591-026-04448-w)
Supplement: Supplementary file 2 [file 41591_2026_4448_MOESM2_ESM.pdf]

nature portfolio

Corresponding author(s): Yin Cao

Last updated by author(s): Apr 17, 2026

Reporting Summary

Nature Portfolio wishes to improve the reproducibility of the work that we publish. This form provides structure for consistency and transparency in reporting. For further information on Nature Portfolio policies, see our Editorial Policies and the Editorial Policy Checklist.

Statistics

For all statistical analyses, confirm that the following items are present in the figure legend, table legend, main text, or Methods section.

n/a	Confirmed	
☐	☒	The exact sample size (*n*) for each experimental group/condition, given as a discrete number and unit of measurement
☐	☒	A statement on whether measurements were taken from distinct samples or whether the same sample was measured repeatedly
☐	☒	The statistical test(s) used AND whether they are one- or two-sided<br>*Only common tests should be described solely by name; describe more complex techniques in the Methods section.*
☐	☒	A description of all covariates tested
☐	☒	A description of any assumptions or corrections, such as tests of normality and adjustment for multiple comparisons
☐	☒	A full description of the statistical parameters including central tendency (e.g. means) or other basic estimates (e.g. regression coefficient) AND variation (e.g. standard deviation) or associated estimates of uncertainty (e.g. confidence intervals)
☐	☒	For null hypothesis testing, the test statistic (e.g. *F*, *t*, *r*) with confidence intervals, effect sizes, degrees of freedom and *P* value noted<br>*Give P values as exact values whenever suitable.*
☒	☐	For Bayesian analysis, information on the choice of priors and Markov chain Monte Carlo settings
☐	☒	For hierarchical and complex designs, identification of the appropriate level for tests and full reporting of outcomes
☒	☐	Estimates of effect sizes (e.g. Cohen's *d*, Pearson's *r*), indicating how they were calculated

Our web collection on statistics for biologists contains articles on many of the points above.

Software and code

Policy information about availability of computer code

Data collection	No software was used.
Data analysis	All analyses were performed using R (version 4.3.3) in RStudio (version 2026.01.2+418.pro1) with packages including tidyverse (v2.0.0), survival (v3.5-7), Publish (v2023.1.17), mgcv (v1.9-1), ggplot (v3.5.2), and BioAge (v0.1.0). All analyses were considered significant with two-sided P <0.05. Figures were generated in RStudio and subsequently refined for presentation using BioRender.com and Adobe Illustrator 2026 (version 30.3).

For manuscripts utilizing custom algorithms or software that are central to the research but not yet described in published literature, software must be made available to editors and reviewers. We strongly encourage code deposition in a community repository (e.g. GitHub). See the Nature Portfolio guidelines for submitting code & software for further information.

Data

Policy information about availability of data

All manuscripts must include a data availability statement. This statement should provide the following information, where applicable:

- Accession codes, unique identifiers, or web links for publicly available datasets
- A description of any restrictions on data availability
- For clinical datasets or third party data, please ensure that the statement adheres to our policy

The data analyzed in this study were obtained from UK Biobank portal (web link: https://www.ukbiobank.ac.uk/, email: ukbiobank@ukbiobank.ac.uk) and All of Us

Research Program portal (https://www.researchallofus.org/, support@researchallofus.org), subject to controlled access. Researchers can apply to access UK Biobank data through the UK Biobank Access Management System (https://www.ukbiobank.ac.uk/enable-your-research/apply-for-access). Access to All of Us data is available via the Researcher Workbench (https://www.researchallofus.org/), following registration, training, and data use agreement requirements.
The GWAS summary statistics used for calculating PRS are available at https://www.ebi.ac.uk/gwas/, with contact email at gwas-info@ebi.ac.uk. The weights used for the PRS are available at PGS Catalog (https://www.pgscatalog.org/; pgs-info@ebi.ac.uk), at https://www.pgscatalog.org/publication/PGP000237/, https://www.pgscatalog.org/publication/PGP000095/, https://www.pgscatalog.org/publication/PGP000148/, https://www.pgscatalog.org/publication/PGP000491/, https://www.pgscatalog.org/publication/PGP000344/.

Research involving human participants, their data, or biological material

Policy information about studies with human participants or human data. See also policy information about sex, gender (identity/presentation), and sexual orientation and race, ethnicity and racism.

Reporting on sex and gender	Sex and gender information was self-reported by participants at baseline in both the UK Biobank and the All of Us Research Program. Sex was used as a biological attribute, while gender identity was not specifically assessed. Models were adjusted for sex to account for potential differences in aging and cancer risk. Additionally, sex differences were considered in the trends of accelerated aging by birth cohort and in the association between accelerated aging and cancer risk. Results are reported disaggregated by sex where relevant.
Reporting on race, ethnicity, or other socially relevant groupings	Race and ethnicity were self-reported by participants in both cohorts. In the UK Biobank, race was categorized as White or Other due to the predominantly European ancestry of the cohort. To control for potential confounding in the UK Biobank, models were adjusted for genetic ancestry (first 10 principal components in UK Biobank) and socioeconomic status (Townsend Deprivation Index in UK Biobank; Social Deprivation Index in All of Us).<br>In the All of Us Research Program, race and ethnicity were categorized as non-Hispanic White, non-Hispanic Black, Hispanic, and Other, reflecting the diverse composition of the cohort. These variables were used to assess disparities in accelerated aging and cancer risk.
Population characteristics	The study population included participants under age 55 at baseline from two large, well-characterized cohorts:<br>1. UK Biobank – Predominantly European ancestry cohort, with extensive genetic, sociodemographic, lifestyle, and health data.<br>2. All of Us Research Program – A diverse U.S. biomedical cohort with EHR-linked data, capturing a broader range of racial/ethnic backgrounds and socioeconomic factors.<br>Key covariates included age, sex, race/ethnicity, BMI, smoking status, alcohol intake, physical activity, and personal and family history of chronic diseases and cancer.
Recruitment	UK Biobank participants were recruited from the general UK population between 2006 and 2010, with voluntary participation following informed consent. The All of Us Research Program recruits participants on an ongoing basis since 2010 from healthcare systems and community outreach efforts across the U.S., ensuring a diverse study population. While self-selection bias may be present, previous studies have demonstrated the generalizability of these cohorts for aging and cancer research.
Ethics oversight	Ethical approval for the UK Biobank study was granted under UK Biobank Application No. 55288. The All of Us Research Program operates under approvals from the Institutional Review Board (IRB) of the All of Us Research Program, overseen by the U.S. National Institutes of Health (NIH). All participants provided informed consent for data collection and research use.

Note that full information on the approval of the study protocol must also be provided in the manuscript.

Field-specific reporting

Please select the one below that is the best fit for your research. If you are not sure, read the appropriate sections before making your selection.

☒ Life sciences ☐ Behavioural & social sciences ☐ Ecological, evolutionary & environmental sciences

For a reference copy of the document with all sections, see nature.com/documents/nr-reporting-summary-flat.pdf

Life sciences study design

All studies must disclose on these points even when the disclosure is negative.

Sample size	No pre-determined sample size calculation was performed for this prospective cohort analysis. Sample sizes were determined by the number of eligible participants available in the UK Biobank and All of Us cohorts after applying predefined inclusion and exclusion criteria. These large population-based cohorts provide sufficient statistical power to detect modest associations. In the UK Biobank, we included 154,169 participants under age 55 at baseline who completed sociodemographic, lifestyle, and health questionnaires, underwent a physical examination, and provided blood samples. In the All of Us Research Program, we included 10,262 participants under age 55 at enrollment with available survey, electronic health record (EHR), and biospecimen data. Sample size adequacy has been validated in prior studies leveraging these cohorts, which have demonstrated sufficient statistical power for similar epidemiological and biomarker analyses.
Data exclusions	Participants with missing or extreme values (>5 SD) for PhenoAge biomarkers, missing covariates critical for analyses (e.g., genetic ancestry, BMI, smoking status), or a cancer diagnosis (except non-melanoma skin cancer) before or within six months of baseline were excluded. Additionally, participants classified as underweight (BMI <18.5 kg/m^2) were excluded to minimize confounding by frailty. In the UK Biobank, 148,317 participants remained after applying these exclusions, and in the All of Us Research Program, 8,935 participants were included in the final analysis. All exclusion criteria were pre-established to ensure data completeness and robustness.

Replication	Reproducibility was assessed using two independent population-based cohorts, the UK Biobank and the All of Us Research Program. Consistent analytical approaches were applied across both cohorts, including PhenoAge estimation, birth cohort analyses, and cancer risk modeling. Key findings were directionally consistent across cohorts, supporting the robustness of the results. All analyses were conducted using standardized and reproducible pipelines.
Randomization	As this was an observational cohort study, participants were not randomized into experimental groups. However, potential confounders, including sociodemographic, lifestyle, and genetic factors, were rigorously adjusted for in multivariable models to mitigate bias.
Blinding	Blinding was not applicable as this study utilized pre-existing cohort data without direct intervention.

Reporting for specific materials, systems and methods

We require information from authors about some types of materials, experimental systems and methods used in many studies. Here, indicate whether each material, system or method listed is relevant to your study. If you are not sure if a list item applies to your research, read the appropriate section before selecting a response.

Materials & experimental systems

n/a	Involved in the study	
☒	☐	Antibodies
☒	☐	Eukaryotic cell lines
☒	☐	Palaeontology and archaeology
☒	☐	Animals and other organisms
☒	☐	Clinical data
☒	☐	Dual use research of concern
☒	☐	Plants

Methods

n/a	Involved in the study	
☒	☐	ChIP-seq
☒	☐	Flow cytometry
☒	☐	MRI-based neuroimaging
